# Data recoverability and estimation for perception layer in semantic web of things

**DOI:** 10.1371/journal.pone.0245847

**Published:** 2021-02-26

**Authors:** Rabia Afzaal, Muhammad Shoaib

**Affiliations:** Department of Computer Science, University of Engineering and Technology Lahore, Lahore, Pakistan; Hankuk University of Foreign Studies, REPUBLIC OF KOREA

## Abstract

Internet of Things (IoT) is the growing invention in the current development of different domains like industries, e-health, and education, etc. Semantic web of things (SWoT) is an extension of IoT that enhance the communication by behaving intelligently. SWoT comprises 7 layered architecture. The perception layer is an important layer for collecting data from devices and to communicate with its associated layer. The data loss at the perception layer is very common due to inadequate resources, unpredictable link, noise, collision, and unexpected damage. To address this problem, we propose a method based on Compressive Sensing which recovers and estimates sensory data from a low-rank structure. The contribution of this paper is three folds. Firstly, we determine the problem of data acquisition and data loss at semantic sensory nodes in SWoT. Secondly, we introduce a compressive sensing based framework for SWoT that recovers the data accurately using low-rank features. Thirdly, the data estimation method is utilized to reduce the volume of the data. Proposed Compressive Sensing based Data Recoverability and Estimation (CS-RE) method is evaluated and compared with the existing reconstruction methods. The simulation results on real sensory datasets depict that the proposed method significantly outperforms existing methods in terms of error ratio and data recoverability accuracy.

## Introduction

The Internet of Things is an emerging technology that provides the fundamental structure for the new generation by connecting things. Internet of Things (IoT)-based systems is expanding nowadays to enhance the production efficiency and quality of our lives. Experts estimate that 50 billion devices will get connected with IoT by Semantic Web of Things (SWoT) is a modern research area that targets to acquire Semantic Web-based technologies with the Internet of Things. It can also be considered as a transformation of the Web of Things (WoT) by fusing semantics. Semantic Web of Things (SWoT) targets the capacity to exchange and utilize data among different ontologies. The larger number of interconnected devices results in scalability, heterogeneity, and various interoperability problems; therefore, an adaptable infrastructure is needed to resolve these issues. Semantic technologies can be an essential enabling technology for sensor networks because they can enhance semantic interoperability and integration. A semantic sensor network can provide the network, its sensors, and the resulting data to be installed, managed, organized, asked, learned, and controlled by high-level specifications. Semantic annotation of sensor information, sensor network management, and services that support sensor data transfer will serve a similar purpose as that adopted by a semantic annotation of Web services. IoT Applications perform data analysis and real-time predictive analytics that need informative automated measurements. IoT-based sensor solutions generally promote interoperability and ubiquitous computing by modifying low-level sensor data into high-level information that is comprehensive to the machines and humans. The challenges in designing inter-domain IoT applications are to combine, reuse, exploit, and interpret sensor data. Federated Interoperable Semantic IoT Testbeds and Applications (FIESTA) are initiatives of SWoT [1]. FIESTA-IoT provides equipment and techniques for assisting IoT based systems [2]. It helps to communicate using semantics-based solutions in an interoperable way.

Semantic Web of Things (SWoT) is a modern research area that targets to acquire Semantic Web-based technologies with the Internet of Things. It can also be considered as a transformation of the Web of Things (WoT) by fusing semantics. Semantic Web of Things (SWoT) targets the capacity to exchange and utilize data among different ontologies [1]. The larger number of interconnected devices results in scalability, heterogeneity, and various interoperability problems; therefore, an adaptable infrastructure is needed to resolve these issues. Semantic technologies can be an essential enabling technology for sensor networks because they can enhance semantic interoperability and integration [3,4]. A semantic sensor network can provide the network, its sensors, and the resulting data to be installed, managed, organized, asked, learned, and controlled by high-level specifications. Semantic annotation of sensor information, sensor network management, and services that support sensor data transfer will serve a similar purpose as that adopted by a semantic annotation of Web services.

The layered architecture of the Semantic Web of Things (SWoT) was introduced by a scholar Amelie and her group members [3,4]. The SWoT architecture consists of 7 layers named as, perception layer, data acquisition layer, persistence layer, knowledge management layer, reasoning layer, knowledge query layer, and application layer. Perception layer is vital for gathering data from devices and communicate with its related layers [5–7].

The SWoT is a great advancement in the technology era and there are various applications of IoT and SWoT such as smart transportation, smart city, smart grid, smart manufacturing, and smart agriculture, etc. It has a vast range of applications in the e-health structure, industries, and education [8]. The applications of IoT and SWoT are shown in S1 Fig.

A SWoT includes many independent elements such as sensors, RFID tags, actuators, computers, and mobile phones, etc. These components can generate and communicate data using procedures to collect, transmit, and store data [9,10]. Sensors mainly collect data from the real world. This data can range from all kinds of environments like temperature, heat, light, motion, humidity, and pressure, etc. However, data collection is mainly affected due to wireless and hardware limitations. A raw dataset has a significant missing data. The amount of missing data grows larger as a wireless system spreads [11]. Therefore, missing data is a fundamental challenge for accurate data reconstruction. An acceptable data compression rate is required in SWoT which cannot be achieved by current techniques without adding distortions [12].

In the perception layer of the IoT and SWoT, the sensor networks play a crucial role. This is because the sensor networks allow us to acquire various kinds of environment information. The perception layer in SWoT behaves like five sense organs for the IoT and SWoT architectures. The perception layer senses, gathers information, and submits the collected information to the higher application layer for intelligent processing via Internet or other transmitted networks. The perception layer is necessary for collecting data and communicating with its associated layers [13]. It consists of three components; sensors, RFID tags, and actuators. The perception layer collects environmental information and communicate with the network. The missing data is compressed, predicted, recovered, and estimated at the perception layer. The sensor nodes at the perception layer in SWoT can gather different kinds of environmental data. It can serve as a bridge between the real world and the digital world [14]. For IoT perception layer, terminal can be a sensor node or a device. The perception layer is also responsible for missing data recovery, data authentication, and access control mechanisms by preventing unauthorized users from accessing the data of sensor nodes in the IoT and SWoT architectures.

Sensors at the perception layer gather the environmental data. The fast growth and deployment of sensor technology are magnifying the current problem of huge data and not enough information. Intelligent sensors should be seamlessly and securely interconnected to facilitate high-level smart applications. Sensor data can be interpreted with semantic data to improve interoperability among heterogeneous sensor networks and to share contextual information essential for circumstances recognition. There are several sensor nodes in the SWoT network that collect the sensory data from the real environment and interact with the other nodes in the network. Sensor nodes in SWoT network are called semantic sensor nodes. The intelligent sensor node in a network is able to perform some processing, collecting sensory data, and interacting with other associated nodes in the network. These sensor nodes are tiny electronic devices that utilize very low energy and they are also adaptive to the real environment. Each sensor node has a specific coverage area for which it can certainly and correctly report the information.

The motivation behind this work is that basic scientific work significantly depends on data reconstruction efficiency and accuracy. The perception layer is very important for all the IoT and SWoT architectures. However, due to limited resources, collision, noise, unpredictable links, and accidental wireless system breakage, data loss occurs during wireless data transmission on the sensor nodes of the perception layer. Furthermore, missing data becomes larger as the network grows. Consequently, data loss becomes a key challenge against accurate data reconstruction. It considerably decreases the recoverability efficiency of a system, the reliability of monitoring applications, and even threaten the life safety in medical diagnosis structures. Therefore, data quality enhancement and data reconstruction are very essential for the reliable e-health structure, smart industries, and education [15]. It is necessary to design useful methods that recover and estimate the missing data efficiently. To the best of our knowledge, none of the approaches proposed in literature includes a combination of prediction, reconstruction, and estimation of data in indoor IoT and SWoT.

The Compressive Sensing (CS) method has broad applications in IoT. This method is used to recover the missing data at sensory nodes by using a small segment of data [16–18]. The CS cannot be directly used for data recovery due to its unique structure so the missing data should follow the Gaussian or pure random distribution. The compressive sensing applications of data reconstruction in WSNs and IoT have been considered recently [19]. However, we are applying CS-based recoverability and estimation method for missing data in SWoT by using low-rank approximations. The benefits of using CS-RE method are as follow.

- An estimation method is used to manage the large amount of data as the data size grows at the sensory nodes over time.
- CS-RE method improves the space utilization of the system.
- CS-RE method selects the optimal data from the overall data at the sensory nodes.
- CS-RE method determines the data estimation problem iteratively.
- The estimation method can be utilized for collecting optimal data rather than storing a complete dataset without missing the significant information.
- A large dimensional dataset is decreased to a small dimensional dataset and then forwarded to the network.
- The estimation method decreases the data transmission and data computation loads.

The semantic sensory nodes at the perception layer collect and process the data from the physical world. Its basic purpose is to identify unique objects and deal with data collection. We introduce a Compressive Sensing based Data recoverability and Estimation (CS-RE) method. It compresses and recovers the data based on CS rather than retransmitting the data in the SWoT network. The estimation method is also used at the semantic sensory nodes to reduce the volume of the data. This improves the space utilization of the system.

The contributions are summarized in three folds:

1. We define the problem of missing data and data recovery at semantic sensory nodes in SWoT. Firstly, we take the benefit of the low-rank feature of raw data to design the data compression and recoverability problem in matrix form.
2. A CS-based framework is then proposed for SWoT that includes the compressive sensing, data transmission, and accurate data recovery at semantic sensory nodes based on the low-rank features.
3. An estimation method is used to manage the large amount of data. This improves the space utilization of the system.

In the next sections, we will introduce a compressive sensing-based data recoverability and estimation method. This method applies the compressive sensing method to recover missing data using a low-rank structure. Related work is discussed in Section II. The proposed method for SWoT is given in Section III. It explains an adaptable data recovery method for SWoT based on compressive sensing and estimation method. The results and comparison of our proposed method with existing methods and techniques are discussed in Section IV. The conclusion and future work is discussed in Section V.

### Background and related work

There are several studies on IoT and SWoT networks for data loss and recovery at sensory nodes. In the latest SWoT network, data integrity is the most essential feature that affects the performance of a system. The SWoT is adopted for various applications like telemetry in dangerous environments, running industrial processes, smart transportation networks, e-health structures, national defense, etc. The problem of missing data at sensory nodes in SWoT has been known for a long time [20]. WSN and IoT are the key technologies of SWoT. These have been studied widely due to the fast growth of microelectronics technology, wireless transmission technology, and embedded computing technology [21].

A perception layer in SWoT consists of many sensory nodes, RFID tags, and actuators distributed in a particular area. The semantic sensory nodes capture and transmit the data. Numerous research studies have concentrated on reconstructing missing data in IoT and wireless sensor networks but data recoverability and estimation are not much discussed in SWoT networks. Also, the recent research works do not discuss the advantage of data compression, reconstruction, and estimation for an indoor SWoT network. Missing data shows notable challenges in accurately recovering the real world data. Thus, it is required to come up with an efficient method to retrieve missing data from inadequate information and further estimate the data to bring down the volume of data. The perception layer architecture, functions, and data handling methods are shown in S2 Fig.

In [22], Haupt examined the compressive sensing for network data in IoT and WSNs. The author analyzed the shared data resources and data sampling in sensor networks. The long-term data collection in large sensor networks is considered which continue till the lifetime of a sensor network. The purpose of this approach is to reconstruct the missing data accurately.

In [23], a novel compressive sensing model is proposed using expander graphs. Maximum a posteriori (MAP) algorithm is introduced for retrieving the compressed data from Poisson predictions. The expander graphs and sensing matrices are used to bound the data reconstruction error. This method can estimate the unknown quantity based on empirical data. The results are validated with experimental illustrations of restoring packet return rates and instant packet counts at a router in a wireless communication network. This method is also efficient but a posterior distribution does not have any simple analytical form.

Chen et al. introduced a Multi Attribute-assistant Compressive Sensing (MACS) algorithm in [24] for accurate data recovery optimization. The authors offered a common sparse decomposition approach to discover the hybrid features between different traits. The two real datasets were used for recovering the multi-attribute datasets.

Cheng et al. [25] introduced an Efficient Data Collection Approach (EDCA) for data collection in wireless sensor networks. EDCA uses the benefit of the low-rank component for achieving low data traffic and large accuracy. EDCA selects the sensor node and time slot for data sampling to reduce the energy consumption of the system. It applies a matrix completion method to retrieve the missing data. EDCA employs the low-rank component of the missing data matrix. It cannot achieve significant accuracy for a very high missing data rate.

Kong et al. [26] examined the environmental information and reported four different characteristics of sensor data; time stability, multi-attribute relationship, space similarity, and low-rank. The authors introduced an Environmental Space-Time Improved Compressive Sensing (ESTI-CS) algorithm for computing and recovering the data loss. ESTI-CS assumed the smallest low-rank estimates of the incomplete raw data matrix. It interpreted the association with spatiotemporal characteristics, and follow the powerful association of multiple attributes from a similar dataset for reliable remodeling accuracy.

The most common convex optimization algorithm is Gradient Projection for Sparse Reconstruction GPSR [27]. This algorithm is introduced to find the sparse solution providing a speedy and reliable completion of data reconstruction in IoT applications. It is extensively applied in compressive sensing data reconstruction. The GPSR algorithm operates in a broad spectrum of applications and does not require application-specific tuning. Although it shows better accuracy but computational complexity is very large.

Least absolute shrinkage and selection operator (LASSO) [28] is the most popular data loss minimization in compressive sensing. It can work better in a wide spectrum of conditions. LASSO is also employed to recover the lost data at sensory nodes. It can handle the multivariate data in different spaces and it is also energy efficient. However, this paradigm is complicated in terms of computations and processing rates.

The Orthogonal Matching Pursuit OMP [29] is also a recovery algorithm for compressive sensing. It is used to handle the non-convex combinational optimization problem. It is a greedy algorithm as it can choose all the notable elements before possibly choosing the wrong ones. The signal or data can be recovered easily but the data recovery is very low. This method is less efficient as compared to other methods but it shows less computational complexity.

A methodology called GRASTA (Grassmannian Robust Adaptive Subspace Tracking Algorithm) is introduced in [30]. This method recovers the missing data in IoT applications based on partial information. GRASTA uses the low-rank features for optimization along with sparse components. GRASTA efficiently estimates the data and reduces space requirements. This method is mostly applied for a small amount of data loss, but a huge volume of data loss shows severe computational overheads.

Various methods have been proposed in the research to handle data loss. The algorithms and approaches discussed above examine the problem of recovering the missing data in WSN and IoT networks. These methods face issues to recover data accurately for different data missing patterns. A method is needed which recovers and estimate the data accurately in real environment for different data missing patterns. This paper concentrates on designing a method that recovers and estimates the sensory data accurately in the presence of data loss at semantic sensory nodes of the perception layer in SWoT.

## Materials and methods

Missing data problems are very common in SWoT [31,32]. The main reasons behind data loss are limited resources, noise, collision, and accidental damage to the wireless system. The semantic sensory nodes collect and process the data from the physical world. A wide range of research work is done to recover the missing data at sensory nodes in SWoT. Compressive sensing is a general approach to retrieve the complete dataset by observing a few samples of data. We introduce a CS-RE method for recovering the missing data instead of retransmitting the data by the semantic sensory nodes. We are using a low-rank structure for accurate data recovery. This method also reduces the volume of the transmitted data and improves the space utilization of the system.

Table 1 shows the notations used in the paper.

**Table 1:** Notations.

Symbol	Description	Symbol	Description
X	Environmental data	*L*	Random matrix
S	Sensory data	R	Max inverse
*B*	Binary index matrix	*N*	Number of nodes
*ϵ*	Error ratio	*t*	Time slot
r	Matrix rank	λ	Tradeoff parameter
Ẍ	Recoverability data	*σ*_*i*_	i-th energy element

### Data acquisition at sensory nodes

According to the Compressive Sensing approach, the data loss in the real dataset can be recovered by using the collected data. Assume, there are N sensory nodes in the given structure and the monitoring period has t time slots, we must acquire N data samples to recover the original signal X. All the sensory nodes need to send data using a wireless network in a given time slot. *X*(*i*, *j*) is the sensory data of node i at time slot j. Here i = 1,2,…‥,N and j = 1,2,……,t. The X signal in a dynamic environment can be represented using Eq (1).

$$\text{X}={\left(\text{x}\left(\text{i},\;\text{j}\right)\right)}_{\text{N}\times \text{t}}$$ (1)

The semantic sensory data is considered for evaluation. We can collect the data for signal S by using M generalized linear functions at t time slots. Here, the signal X can be recovered by using *M* × *N* matrix B. The size of M is very much smaller than the size of N. Hence, the relation between X and S can be defined using Eq (2).

S=B•X (2)

We call the matrix B as binary index matrix (BIM). It is indicated by a *N* × *t* matrix that shows the missing data points. BIM is represented using Eq (3).

$$B={\left(b\left(i,j\right)\right)}_{N\times t}=\{\begin{array}{c} 0\;if\;x\left(i,j\right)is\;missing\\ \;1\;otherwise. \end{array}$$ (3)

The principal idea behind compressive sensing in SWoT is that many physical-world signals or datasets show some redundancy. It employs the last information for acquiring and reconstructing the signal or dataset under consideration. Compressive sensing employs the vectors form of data or signals. A matrix approach can organize this data into vectors. The matrix X is a real matrix as some part of the data matrix is inherent. We have used the low-rank structure of the matrices for recovering and fitting the required data accurately [33].

### Problem statement

Data recoverability is to reconstruct the real environmental data or signal using the sensory data at the perception layer in SWoT. The data is recovered by introducing the missing contents in a sensory matrix for real data approximation. The recoverability matrix denoted by Ẍ should approximate the original signal X as closely as possible.

$$\begin{array}{c} Objective:\;min{\left\|X-Ẍ\right\|}_{F},\\ Subject\;to:\;S \end{array}$$ (4)

Where ${\left\|\cdot \right\|}_{F}$ is a Frobenius norm. It is applied to estimate the error among the matrix X and Ẍ. We can calculate X by using Frobenius norm such as: ${\left\|\text{X}\right\|}_{\text{F}}=\sqrt{\sum_{\text{i,j}}\text{x}\left(\text{i},\text{j}\right){)}^{2}}$

The main objective of data recoverability problem is to reduce the absolute error. We have used an error ratio metric for measuring the recoverability error in different situations and fit the missing data accurately. The error ratio metric is shown in Eq (5).

$$\epsilon =\frac{\sqrt{\sum_{i,j:b\left(i,j\right)=0}{\left(x\left(i,j\right)-Ẍ\left(i,j\right)\right)}^{2}}}{\sqrt{\sum_{i,j:b\left(i,j\right)=0}{\left(x\left(i,j\right)\right)}^{2}}},$$ (5)

The errors should be measured only on the missing values so ***ϵ*** is determined when the missing value exists in the dataset. Here, the condition b(i, j) = 0 in Eq (5) shows that only errors on the missing data are counted.

### Data loss in sensory nodes

In this section, we investigate the data loss in real environmental datasets. We employ real-world datasets from the Intel Berkeley Indoor project [34], GreenObs project [35], and Ocean Sense project [36] for simulations. There exists heavy data loss in these real datasets.

We use two data processing patterns to check the efficacy of our proposed method accurately. These are called random missing patterns and consecutive missing patterns. Firstly, we receive the entire raw dataset and then introduce the artificial missing using two patterns.

1. **Random Missing Pattern (RMP):** This is a very simple missing pattern. The purpose of this pattern is to drop the random sensory elements at a random time and therefore the data is missing in this pattern.
2. **Consecutive Missing Pattern (CMP):** In this pattern, if a node begins to loose from a specific time slot, it can miss all the data after the node where the data loss begins. This type of data loss happens due to damaged sensory nodes.

The two data missing patterns are shown in S3 and S4 Figs.

### Low-rank structure

The low-rank structure is used with compressive sensing to recover the massive data loss [37]. In this paper, we focus on compressive sensing recovery and estimation methods for low-rank matrices to recover and fit the lost data accurately. We can formulate the missing data recoverability problem into the matrix rank minimization problem. The main objective is to reconstruct all the components using a few observed components of a specific matrix. It is explained by following the rank minimization problem.

Singular Value Decomposition (SVD) is the fundamental mechanism for generating low-rank matrix approximations. Recoverability matrix is presented by Ẍ = (ẍ(i, j))_N×t_ and a *N* × *t* real data matrix can be disintegrated into three matrices.

$$\text{X}={\text{U}\Sigma \text{V}}^{\text{T}}=\sum_{\text{i}=1}^{\min(\text{N},\text{t})}\sigma_{\text{i}}{\text{u}}_{\text{i}}{\text{v}}_{\text{i}}^{\text{T}}$$ (6)

Here, U presents *N* × *N* unitary matrix and V presents *t* × *t* unitary matrix. *V*^*T*^ shows the transpose of V and *∑* presents a *N* × *t* diagonal matrix. *σ*_*i*_ is also a unique value that shows the i-th energy element. Typically, the unique values are sorted in Σ such as *σ*_*i*_ ≥ *σ*_*i+*1_ and *i* = 1,2,…‥min(*n*,*t*). Here, min(*n*,*t*) is the total number of unique values. Thus, we can say the total energy is the sum of all the unique values $\sum_{\text{i}=1}^{\min(\text{N},\text{t})}\sigma_{\text{i}}$. Ẍ is perceived as the best r-rank approximation using the Frobenius norm ${\left\|\cdot \right\|}_{F}$ for the estimated errors. Therefore, the SVD mechanism presents the simplest solution for r-rank minimization.

$$\begin{array}{l} \text{Objective}\;\text{Minimize}\;{\left\|\text{X}-Ẍ\right\|}_{\text{F}}\\ \text{Subject}\;\text{to}\;\text{rank}\left(Ẍ\right)\leq \;\text{r} \end{array}$$ (7)

The rank of any matrix is expressed by ’r’ which is a unique non-zero value. The matrix will be considered as low rank for *r* << min(*n*,*t*). S5 Fig shows the low-rank structure for two raw environmental datasets containing indoor-temperature and indoor-light. Here, the x-axis depicts the i-th unique value normalized by min(*n*,*t*). Y-axis shows the summation of first i-th unique value normalized by max(*σ*_i_).

### Compressive sensing

We will apply the compressive sensing based method to a low-rank structure mathematically. We have exhibited the low-rank structure in the real environmental data sets and propose a compressive sensing based method to recover and estimate the missing data from semantic sensory nodes. Here, we introduce the CS theory to recover the missing data accurately.

The purpose of solving the recoverability problem is to measure Ẍ. As it is mentioned in Eq 6 that any matrix can be disintegrated by SVD into $\sum_{i=1}^{min(\text{N},\text{t})}\sigma_{i}u_{i}v_{i}^{T}$. Through the inverse of the SVD matrix, we can also build an r-rank approximation Ẍ by only considering the largest unique values of ‘r’ and discarding the others. Then Eq (6) can be inversed for the r-rank approximation Ẍ as:

$$\sum_{\text{i}=1}^{\text{r}}\sigma_{\text{i}}{\text{u}}_{\text{i}}{\text{v}}_{\text{i}}^{\text{T}}=Ẍ$$ (8)

This recoverability matrix Ẍ is recognized as the best r-rank approximation as it reduces the error estimated by the Frobenius norm. However, we cannot find the optimal Ẍ directly through this process because the original matrix X and rank is not known in advance.

$$\begin{array}{l} Objective:\;\min\left(rank\left(Ẍ\right)\right)\\ Subject\;to:\;S=B\;•Ẍ \end{array}$$ (9)

The recoverability matrix should be closer to the sensory matrix and it should also have a low-rank structure. We can also use the benefit of the SVD-like factorization that can adjust the Eq (8) as:

$$Ẍ={\text{U}\Sigma \text{V}}^{\text{T}}={\text{LR}}^{\text{T}}$$ (10)

We put the values of L = *U∑*^1/2^ and R = *VΣ*^1/2^ to Eq (10) and we can determine the minimization problem based on compressive sensing method in [38]. Thus, we are required to find the matrix L and R to minimize the sum of their Frobenius norms and get the lowest possible rank.

$$\begin{array}{l} Minimize\;{\left\|L\right\|}_{F}^{2}+\|LR^{T}\|{}_{F}^{2}\\ Subject\;to\;S=B\;•\left(LR^{T}\right) \end{array}$$ (11)

The real data mostly estimate the low-rank solutions but it cannot find the accurate low-rank values. The errors and noises at the sensor nodes introduce the over-fitting issue. So we will not solve the Eq (11) directly and solve this equation by applying the Lagrange multiplier method to mitigate these obstacles.

$$Minimize\;\|B•LR^{T}-{\left\|S\right\|}_{F}^{2}+⋋\left({\left\|L\right\|}_{F}^{2}+\|R^{T}\|{}_{F}^{2}\right)$$ (12)

Here, the λ is called as Lagrange multiplier. It permits adjustable tradeoff among the r-rank minimization and accuracy fitness. This method gives the low-rank approximation. Therefore, the R and L matrices are used for the rank optimization problem under the tuning of λ to fit the measured data and achieve a low rank.

We can use missing data recoverability method to store the optimal data instead of storing the complete dataset without wasting important information at the perception layer in SWoT. It offers fast data processing and reliable data recoverability at sensory nodes.

### Design optimization

The two parameters are very important in the CS-RE method, rank ’r’ and tradeoff coefficient λ. The estimation and approximation quality of X greatly depend on these two parameters. Our proposed method is used to derive the optimal values of ’r’ and λ. Here, the error ratio metric is used for fitting the missing data accurately. We can obtain the optimal parameters when the fitness is achieved. The methodology of our proposed CS-RE method is described in S6 Fig.

### Data estimation

The data size grows at the sensory nodes over time. Thus, more space is needed for data collection [39]. We need to bring down the size of data to enhance the space utilization of the network. A data estimation method is needed which selects optimal data from the overall data at the sensory nodes. Our proposed CS-RE method solves this problem by estimating the optimization problem. CS-RE method determines the data estimation problem iteratively. We initialize L randomly to calculate the value of R. Here the value of i is from 1 to t. This is a composite of multi-standard least-square problems. Once we get the value of *R*^*T*^, L can also be recomputed by putting the value of *R*^*T*^. It is observed in Section 4 that the L*R*^*T*^ mostly converge up to five iterations. This joint re-computing procedure continues until the optimal value is obtained. We compute the inverse matrix for the best optimization and estimation solution in Eq (13) such as:

$$\left[\begin{array}{c} B•\left(LR^{T}\right)\\ \sqrt{⋋}R^{T} \end{array}\right]=\left[\begin{array}{c} \;S\\ 0 \end{array}\right]$$ (13)

The equation can be rewritten for ith values in given in Eq (14).

$$\left[\begin{array}{c} Diag(B_{\left(i\right)}\left(LR^{T}{}_{\left(i\right)}\right)\\ \sqrt{⋋}R^{T}{}_{\left(i\right)} \end{array}\right]=\left[\begin{array}{c} \;S_{\left(i\right)}\\ 0 \end{array}\right]$$ (14)

The estimation method can be utilized for collecting optimal data rather than storing a complete dataset without missing the significant information. It provides fast data processing at the sensory nodes and sends estimated data to the network. These data recoverability and estimation methods are applied to the perception layer of SWoT.

A large dimensional dataset is decreased to a small dimensional dataset and then forwarded to the network. The estimation method decreases the data transmission and data computation loads.

A low-rank data structure, data compression, recoverability, and estimation are analyzed together in this paper. We merge these methods to enhance the efficiency of the system. The compressive sensing method is applied to the semantic sensory nodes of the SWoT network. When the missing data recoverability is completed, the sensory nodes estimate the complete dataset through a data subset employing the data estimation method. It transmit the estimated data for better space utilization. Algorithm 1 shows the detailed pseudo-code of our proposed CS-RE method.

### Algorithm 1: CS-RE algorithm

***Input***:

 *N: No*. *of sensory nodes*

 *t:* *time slots*

*X*_N×t_: *Real Data Matrix*

*B*_N×t_: *Binary Index Matrix*

*r* = *rank optimization*

*λ* = *Langrange multiplier*

***Output***:

Ẍ_N×t_: *Recoverability Data Matrix*

*Main Procedure*:

*L* ← *randomMatrix;*

*for* 1 *to maxIterations do*

 *R ← maxInverse*(*B*,*S*,*λ r*, *R*)

 *L ← maxInverse*(*B*^*T*^,*R*^*T*^,*λ r*, *S*^*T*^)

 $v\leftarrow Minimize\;\|B•LR^{T}-{S\|}_{F}^{2}+⋋\left({\left\|L\right\|}_{F}^{2}+\|R^{T}\|{}_{F}^{2}\right)$

 $if\;v<\ddot{v}\;then$

  $\ddot{L}\leftarrow L\;and\;\ddot{R}\;\leftarrow R$

   *endif*

*end* *for*

$\ddot{X}\leftarrow \ddot{L}{\ddot{R}}^{T}$

*return Ẍ*;

  //this is the recoverability solution matrix

*data estimation* ← *myInverse*(*B*,*S*,*λ*,*L*,*R*)

*for i* = 1 *to t do*

${\ddot{X}}_{\left(i\right)}\leftarrow \;[Diag\left(B_{\left(i\right)}\left(LR^{T}{}_{\left(i\right)}\right);\sqrt{⋋}R^{T}{}_{\left(i\right)}\right]$

*end* *for*

*return Ẍ*_(i)_

  //estimated solution

The real environmental datasets and different data loss patterns are used for accurate analysis of the proposed method. The low-rank structure is made for missing data problem and compressive sensing is applied for accurate recoverability. The data estimation decreases the data samples that results in reducing the communication over the network.

## Results and discussion

As we have discussed earlier, we have employed the real environmental data from the Intel Berkeley indoor [34], GreenOrbs project [35] and Ocean Sense project [36] for the simulations and results. Intel data was collected in a laboratory where the Mica2dot sensors were used to collect the temperature, light, and humidity datasets. Each sensory node reported the data once every 30 seconds. Finally, this data was merged into one large dataset. We choose 52 sensory nodes and 300 time slots from Intel indoor dataset, hence N = 52 and t = 300. S7 Fig exhibits the sensor positions in the Intel Berkeley Research Lab [34].

GreenOrbs project [35] is a real wireless sensor network application for forest monitoring from 2008 to the present. Almost 450 TelosB nodes are distributed on the Tianmu Mountain in China and collect light, humidity, and temperature once every 10 minutes.

Ocean Sense project [36] was carried out by the Ocean University in China. This dataset comprises 20 TelosB nodes used in the sea of Taipingjiao in China from 2007 to the present, observing an area of 300m×100m. All sensing nodes record temperature and light data every 2 minutes.

The simulations are run on the MATLAB employing a desktop computer with 32 GB RAM and 3.0-GHz Intel i7 CPU. As the complete sensory dataset X is required for error ratio computation and accurate data recoverability, we adopt two pre-processed datasets of indoor-temperature and indoor-light for our experimental results using two datasets.

Our proposed method is compared with four existing methods for missing data interpolation and data recoverability. Two types of environmental data such as indoor-temperature and indoor-light is selected from three datasets for error ratio computation. To check the validity of our proposed method CS-RE, we have selected standard data reconstruction methods for comparison. These methods are ESTI-CS [26], GPSR [27], LAASO [28], and OMP [29]. We have selected similar parameters λ and rank r (λ = 0.15 and r = 10) for all methods and datasets. The basic simulation process is to take the raw signal dataset X and generate a Binary Index Matrix B for two-loss patterns. Then calculate the sensory data S by using the equation S = B • X. All the methods under consideration are tested by using the sensory data matrix S as input and produce the recoverability matrix ***Ẍ*** accordingly. The error ratio is then calculated to check the accuracy of the proposed method and methods under consideration. Finally, the error ratios, recoverability error, and reconstruction accuracy of all methods are compared for performance evaluation in terms of indoor-temperature and indoor-light of Intel, GreenOrbs, and Ocean sense datasets.

S8 Fig shows the basic comparison of our proposed CS-RE method with the ESTI-CS, GPSR, LASSO, and OMP methods for recovering the real data in indoor temperature. The X-axis shows the data loss. S9 Fig shows the basic comparison of our proposed CS-RE method with the ESTI-CS, GPSR, LASSO, and OMP methods for recovering the real data in indoor light. The results are computed by using the data from Intel Berkeley Research Laboratory for indoor temperature and indoor-light. It is observed that our proposed method can recover the missing data with ≤ 7% error ratio and the ESTI-CS method depicts the error ratio of about 18%. GPRS is very close to 25% and the error ratios for LAASO and OMP are more than 45%. Our proposed method CS-RE outperforms approaches under consideration in the case of indoor-light. However, the benefit is less significant as compared to indoor-temperature because the indoor-temperature variation has prominent features, and the indoor light variations are largely affected by indoor-temperature.

S10 and S11 Figs show the comparison of our proposed CS-RE method with the ESTI-CS, GPSR, LASSO, and OMP methods for recovering the real data in temperature and light using the GreenOrbs project dataset. Our proposed method CS-RE outperforms approaches under consideration in the case of temperature and light using the GreenOrbs project dataset. The error ratio with Intel dataset is better than the GreenOrbs dataset because in GreeOrbs project the sensors are deployed in the forest area and many shadows disturb the data integrity and stability and in Intel Indoor dataset, the environmental changes are smooth.

S12–S14 Figs shows the histograms of our proposed and existing methods with random missing pattern. We have used 30%, 40%, and 64% data loss from the GreenOrbs project dataset, Intel dataset, and Ocean Sense dataset respectively. In RMP, the data is missing randomly and showing an overall 30% loss in simulation for GreenOrbs project dataset. Most of the methods do not perform well in this loss pattern as the real environmental variations in terms of temperature and light are not smooth because of forest area. The error ratio for the proposed CS-RE method is almost 20% for 30% RMP data loss in the dataset. In RMP, the data is missing randomly and showing an overall 40% loss in simulation for Intel Indoor dataset. Most of the methods perform well in this loss pattern as the real environmental variations in terms of indoor-temperature and light are very smooth. The error ratio for the proposed CS-RE method is almost 5% for 40% RMP data loss in the dataset. In RMP, the data is missing randomly and showing an overall 64% loss in simulation for Ocean sense project dataset. Most of the methods perform well in this loss pattern as the real environmental variations in terms of temperature and light are also smooth. The error ratio for the proposed CS-RE method is almost 9% for 64% RMP data loss in the dataset.

S15 and S16 Figs shows the comparison histograms of our proposed and existing methods with consecutive missing pattern using the Intel dataset and GreenObs project dataset respectively. Even for the indoor light, our CS-RE method performs much better than the other approaches using Intel Indoor dataset. The initial points of data loss are random in CMP and it loses all elements after origin points. In CMP, the overall data loss is also 40% for Intel dataset and 30% for GreenOrbs dataset in simulations. For CS-RE method, the results are moderate for CMP for both datasets because the time optimization does not affect much as elements dropped in every time of a node. In summary, proposed method CS-RE outperforms ESTI-CS, GPSR, LAASO, and OMP for both datasets.

We compute the recoverability error using the CS-RE method and other methods under consideration for Intel Indoor dataset and GreenOrbs project dataset. The recoverability error is the error between the raw data and the received corrupted data at the sensory nodes. In S17 and S18 Figs, we compare the reconstruction errors for CS-RE and existing methods using the Intel dataset and GreenObs dataset. The results depict that the recoverability error for the CS-RE model is improved by 3% over the ESTI-CS method using Intel Indoor dataset. Although this improvement is not high but it plays an important role in avoiding the data retransmission from semantic sensory nodes. We also compute the recoverability error in the second simulation using the CS-RE method for GreenOrbs project dataset. We compare the reconstruction error for CS-RE and existing methods and the results depict that the recoverability error for the CS-RE model is better than other methods. The results for GreenOrbs dataset are not much better because of the shadows in the forest. It is concluded that the CS-RE method recovers the missing data better than the approaches under consideration for both datasets.

S19 and S20 Figs depicts the reconstruction accuracy of the CS-RE method as compared to ESTI-CS, GPSR, LAASO, and OMP using the Intel and GreenObs datasets. The simulation results show that our proposed CS-RE method outperforms the existing methods under evaluation. It is observed that our proposed CS-RE method recover the missing data better than other methods for high data loss rates regarding both datasets.

The main reason behind this significant gap is that other methods are designed for data classification and prediction, unlike the CS-RE method and these methods only consider the compressive sensing for data recoverability. But our proposed CS-RE method operates with the real environmental data and also uses the minimum low-rank data structure for more accuracy. CS-RE computes the recoverability dataset using the compressive sensing approach for both datasets. It further uses estimation method for a more reliable and comprehensive data reconstruction. The CS-RE takes lesser execution time for simulations due to estimation method.

To summarize, the proposed CS-RE method outperforms the other tested algorithms on the dataset obtained from Intel Indoor dataset, GreenOrbs project, and Ocean sense project considering the two patterns of data missing. Overall, CS-RE obtain lower recoverability error that can be used in almost all tested datasets with different loss ratios. LASSO and OMP produce almost similar but the poor error ratio performance because of poor resource allocation. Both of GPSR and ESTI-CS are better than LASSO and OMP but still worse than CS-RE method. Especially, at the high data loss cases, CS-RE exhibits an evident advantage over other algorithms. Specifically, the proposed algorithm provides better results under the random missing pattern than the consecutive missing pattern for both datasets. In both data sets, CS-RE can successfully achieve an environment reconstruction with almost 10% error for high data loss cases. The wireless channel loss models and malicious attack in extreme cases can produce continuous loss of data or faults, which cannot be reconstructed by the CS-RE method. The tradeoff between the computation time and accuracy in environment can also be improved. As a work in the near future, we will look for other data reconstruction schemes for those extreme cases. CS-RE method cannot perform well when the data missing rate is more than 60% with consecutive missing patterns.

## Conclusion and future work

We have studied the environmental data loss and data recoverability problems in SWoT. We verified the huge data loss in real Intel indoor data set and used the real sensory dataset for indoor-temperature and indoor-light. We used Compressive Sensing approach to recover the lost data instead of retransmitting the data on the sensory nodes. We proposed the CS-RE method to compress, recover, and estimate the missing data by using low-rank features. Our proposed method combined the benefits of compressive sensing and estimation methods for a low-rank structure in SWoT networks. The experimental evaluations illustrated that the CS-RE method outperforms existing data reconstruction methods. The CS-RE method can achieve a less than 10% error ratio with high missing data in the real dataset. The CS-RE also uses the estimation method for collecting optimal data to reduce the communication and computational overheads in SWoT networks. In the future, the tradeoff among the computational time and reconstruction accuracy in real environmental data can be calculated to further improve the efficiency of the system.

## Supporting information

S1 Fig IoT and SWoT applications in the current technology era. Figure shows applications of IoT and SWoT in the technology such as smart homes, smart transportation, smart grid stations, smart city, smart wearable’s, smart homes and smart manufacturing. The other applications are in education, industries and hospitals.
(PNG)

S2 Fig Perception layer architecture, functions, and data handling methods. The figure shows the architecture and functions of the perception layer. A perception layer in SWoT comprises of many sensory nodes, RFID tags, and actuators distributed in a particular area.
(PNG)

S3 Fig Random Missing Pattern (RMP). This figure shows the random missing patterns in sensed data. This is a very simple data missing pattern. The elements in dataset are dropped randomly at random time in this pattern.
(PNG)

S4 Fig Consecutive Missing Pattern (CMP). This figure shows the consecutive missing patterns in sensed dataset. In this particular pattern, if a node begins to loose from a specific time slot, it would drop all the elements after the node where data loss starts.
(PNG)

S5 Fig Low-rank features of real environmental data. This figure shows the low rank structure of two raw environmental dataset having indoor temperature and indoor light. The x-axis shows the ith unique value and the y-axis shows the maximum normalized value. The low rank structure is used here with compressive sensing method to recover the massive data loss.
(PNG)

S6 Fig Methodology for proposed CS-RE. It shows the overall methodology of the proposed CS-RE method. The low rank structure is used with compressive sensing method for recovering data loss.
(PNG)

S7 Fig Sensor locations in Intel Indoor research laboratory. This figure shows the sensor locations of Intel Indoor research laboratory. The sensors were used to collect the temperature, light, and humidity datasets.
(PNG)

S8 Fig Error ratio comparison in basic data loss for Indoor-temperature with Intel dataset. The figure shows the error ratio comparison of out CS-RE method with ESTI-CS, GPSR, LASSO, and OMP considering indoor temperature from Intel dataset.
(PNG)

S9 Fig Error ratio comparison in basic data loss for Indoor-light with Intel dataset. This figure shows the error ratio comparison of out CS-RE method with ESTI-CS, GPSR, LASSO, and OMP considering indoor light from Intel dataset.
(PNG)

S10 Fig Error ratio comparison in basic data loss for forest-temperature with GreenOrbs dataset. This figure shows the error ratio comparison of our CS-RE method with ESTI-CS, GPSR, LASSO, and OMP considering forest temperature from GreenOrbs project.
(PNG)

S11 Fig Error ratio comparison in basic data loss for forest-light with GreenOrbs dataset. This figure shows the error ratio comparison of our CS-RE method with ESTI-CS, GPSR, LASSO, and OMP considering forest light from GreenOrbs project.
(PNG)

S12 Fig Error ratio comparison performance for random missing pattern with Intel dataset. The figure shows the comparison histogram of random missing pattern using Intel dataset with 40% data loss.
(PNG)

S13 Fig Error ratio comparison performance for random missing pattern with Ocean sense dataset. The figure shows the comparison histogram of random missing pattern using Ocean sense project dataset with 64% data loss.
(PNG)

S14 Fig Error ratio comparison performance for random missing pattern with GreenOrbs dataset. This figure shows the comparison histogram of random missing pattern using GreenOrbs project dataset with 30% data loss.
(PNG)

S15 Fig Error ratio comparison performance for consecutive missing pattern with Intel dataset. This figure shows the comparison histogram of our proposed methods with existing methods using consecutive missing pattern in Intel indoor dataset. Overall data loss is 40%.
(PNG)

S16 Fig Error ratio comparison performance for consecutive missing pattern with GreenOrbs dataset. This figure shows the comparison histogram of our proposed methods with existing methods using consecutive missing pattern in GreenOrbs project dataset. Overall data loss is 30% here.
(PNG)

S17 Fig Recoverability error with Intel dataset. This figure shows the comparison of our proposed method with other methods in terms of reconstruction errors using Intel indoor dataset.
(PNG)

S18 Fig Recoverability error with GreenOrbs dataset. This figure shows the comparison of our proposed method with other methods in terms of reconstruction errors using GreenOrbs project dataset.
(PNG)

S19 Fig Accuracy with data missing rate using Intel dataset. This figure shows the reconstruction accuracy of our proposed method as compared to other methods under consideration using Intel indoor dataset.
(PNG)

S20 Fig Accuracy with data missing rate using GreenOrbs dataset. This figure shows the reconstruction accuracy of our proposed method as compared to other methods under consideration GreenOrbs project dataset.
(PNG)

S1 File (ZIP)
