# Supplementary figures and images for: Data recoverability and estimation for perception layer in semantic web of things
Source: PLoS One. 2021 Feb 26;16(2):e0245847. doi: 10.1371/journal.pone.0245847 (PMC7909669; doi:10.1371/journal.pone.0245847)

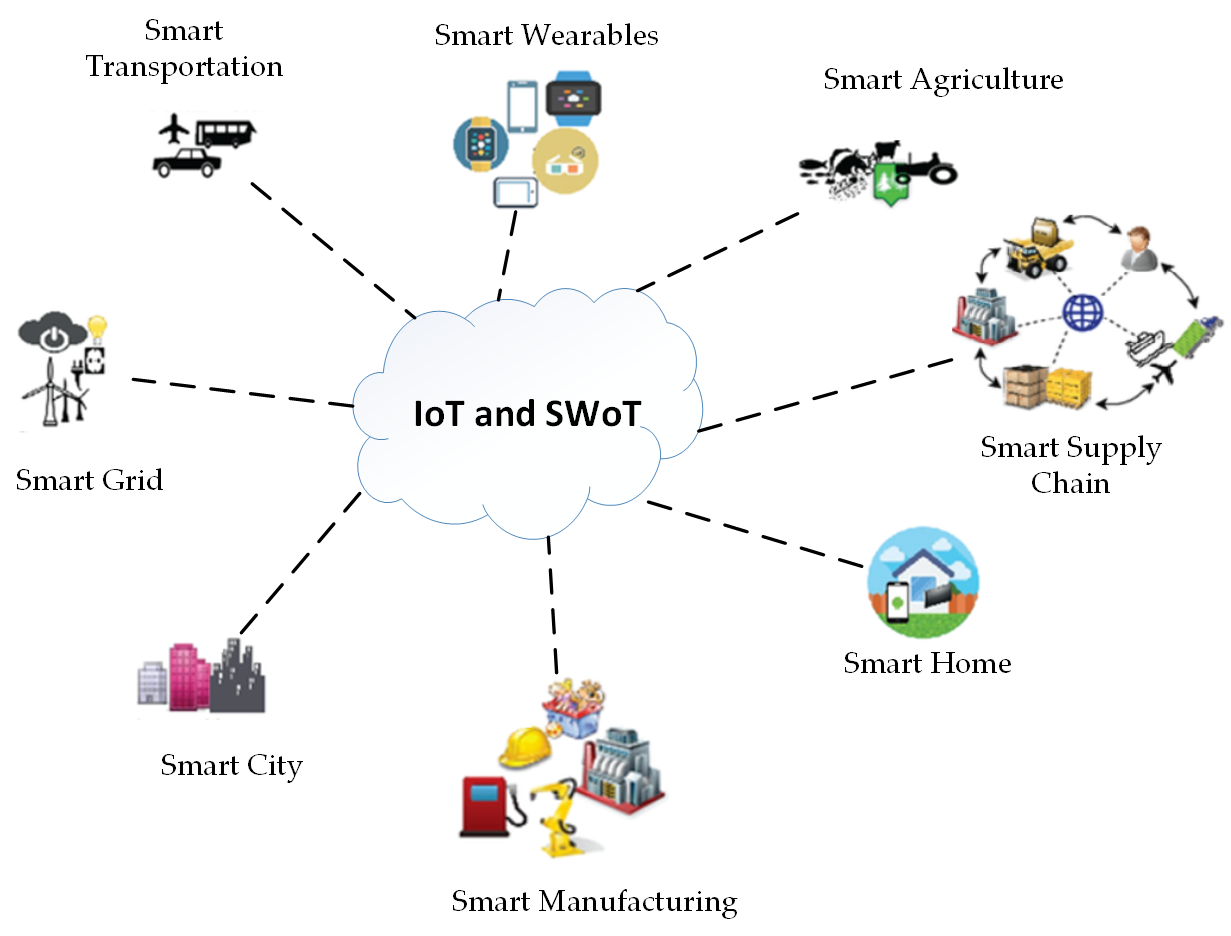

Supplement: S1 Fig — Figure shows applications of IoT and SWoT in the technology such as smart homes, smart transportation, smart grid stations, smart city, smart wearable’s, smart homes and smart manufacturing. The other applications are in education, industries and hospitals. (PNG) [file pone.0245847.s001.png]

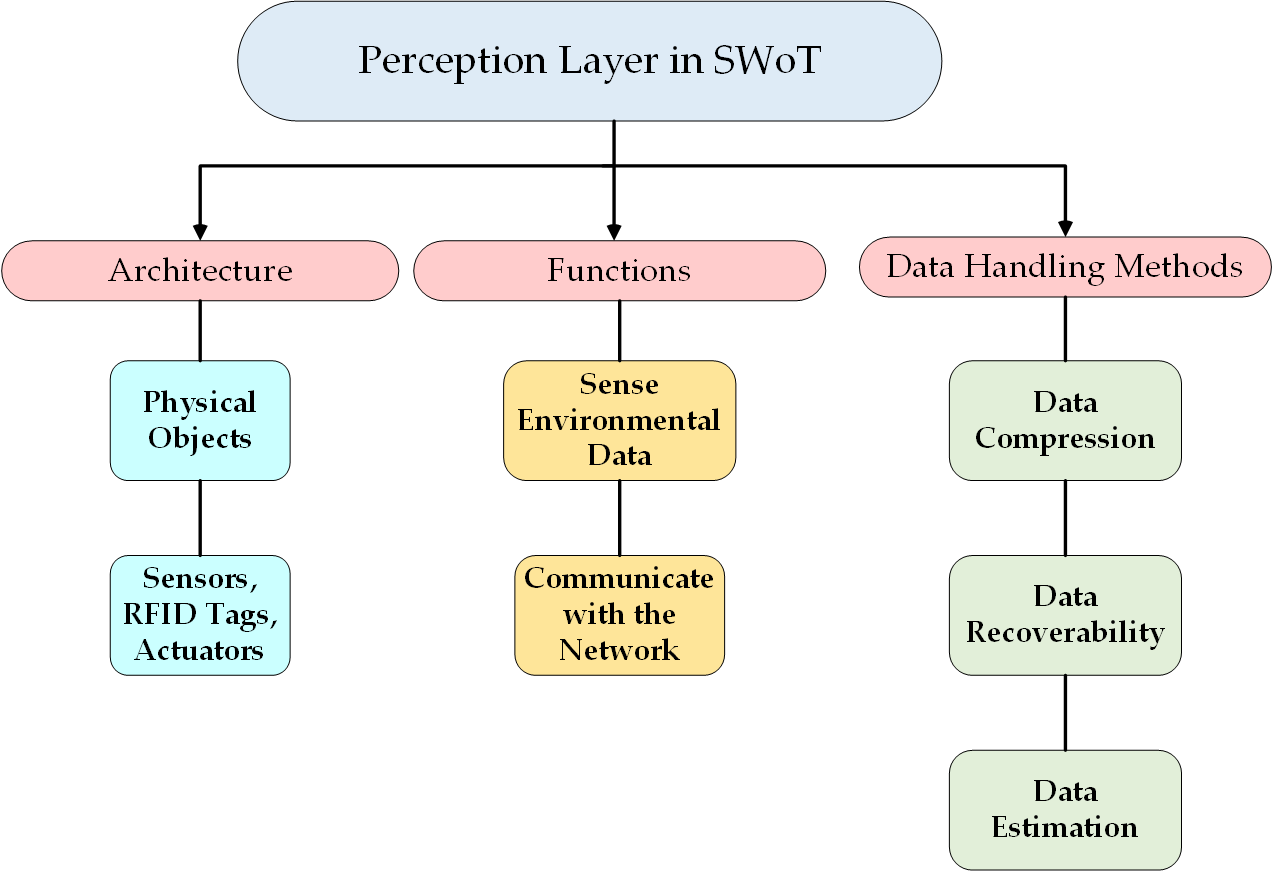

Supplement: S2 Fig — The figure shows the architecture and functions of the perception layer. A perception layer in SWoT comprises of many sensory nodes, RFID tags, and actuators distributed in a particular area. (PNG) [file pone.0245847.s002.png]

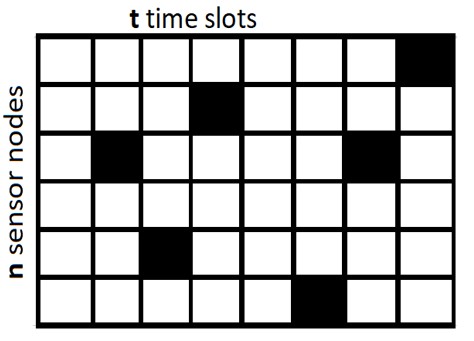

Supplement: S3 Fig — This figure shows the random missing patterns in sensed data. This is a very simple data missing pattern. The elements in dataset are dropped randomly at random time in this pattern. (PNG) [file pone.0245847.s003.png]

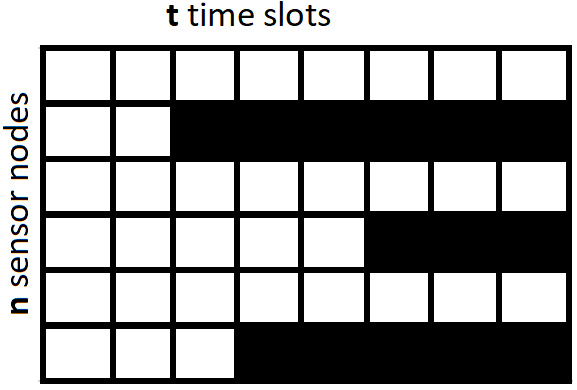

Supplement: S4 Fig — This figure shows the consecutive missing patterns in sensed dataset. In this particular pattern, if a node begins to loose from a specific time slot, it would drop all the elements after the node where data loss starts. (PNG) [file pone.0245847.s004.png]

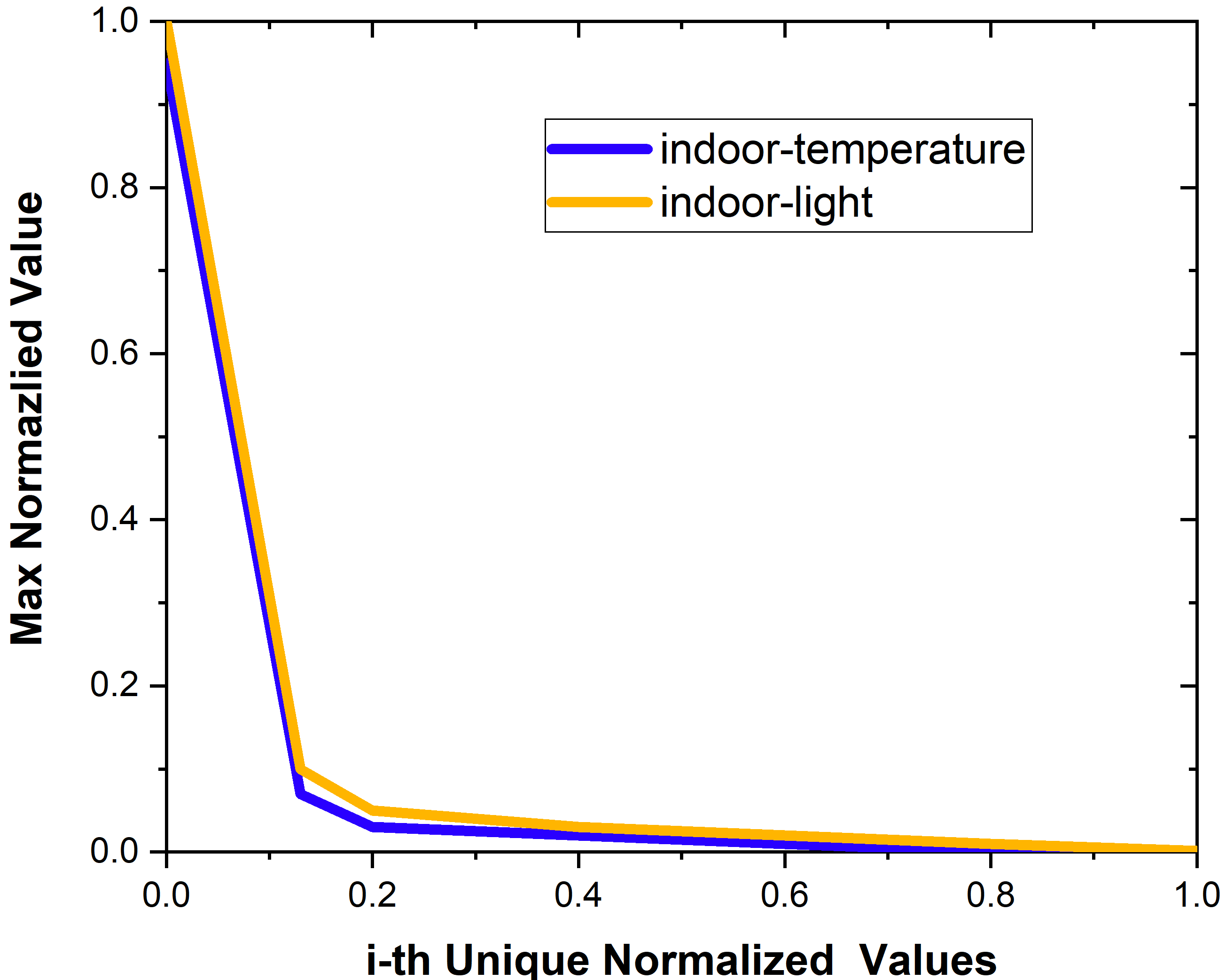

Supplement: S5 Fig — This figure shows the low rank structure of two raw environmental dataset having indoor temperature and indoor light. The x-axis shows the ith unique value and the y-axis shows the maximum normalized value. The low rank structure is used here with compressive sensing method to recover the massive data loss. (PNG) [file pone.0245847.s005.png]

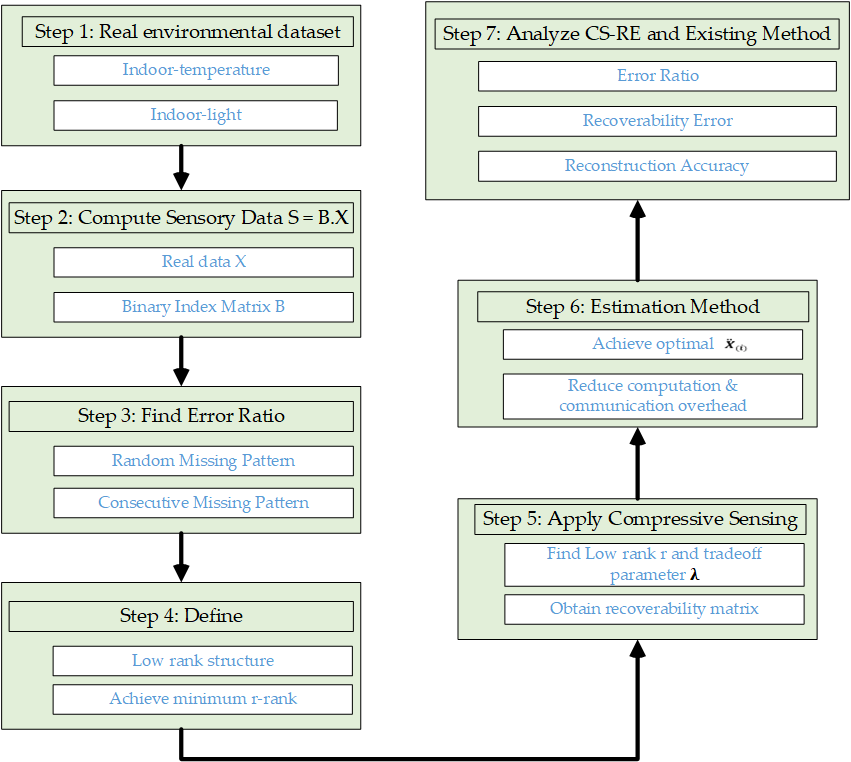

Supplement: S6 Fig — It shows the overall methodology of the proposed CS-RE method. The low rank structure is used with compressive sensing method for recovering data loss. (PNG) [file pone.0245847.s006.png]

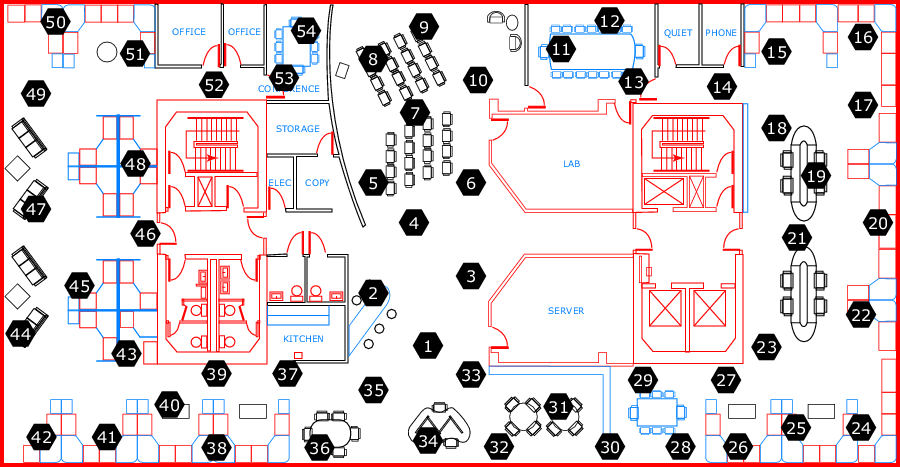

Supplement: S7 Fig — This figure shows the sensor locations of Intel Indoor research laboratory. The sensors were used to collect the temperature, light, and humidity datasets. (PNG) [file pone.0245847.s007.png]

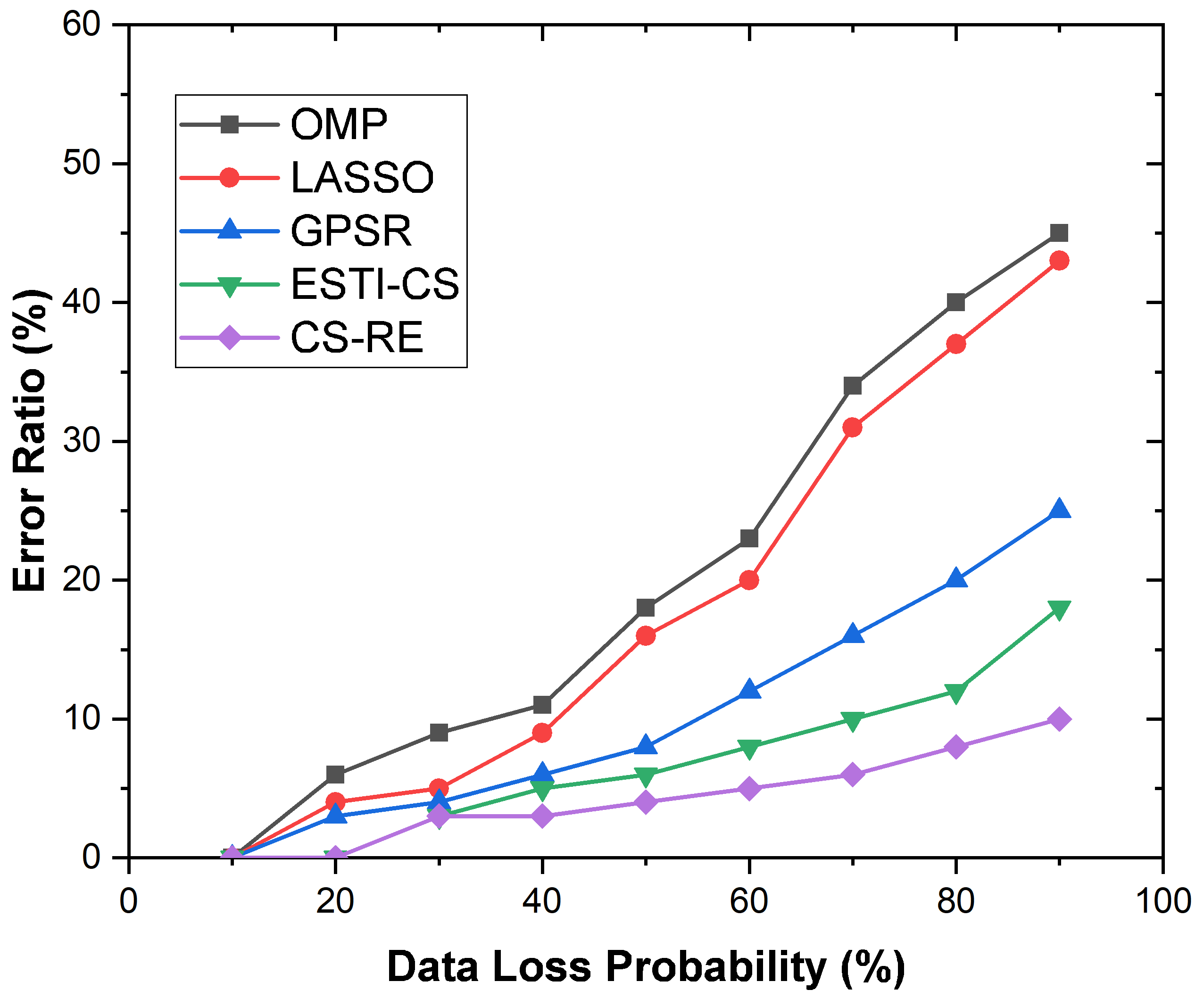

Supplement: S8 Fig — The figure shows the error ratio comparison of out CS-RE method with ESTI-CS, GPSR, LASSO, and OMP considering indoor temperature from Intel dataset. (PNG) [file pone.0245847.s008.png]

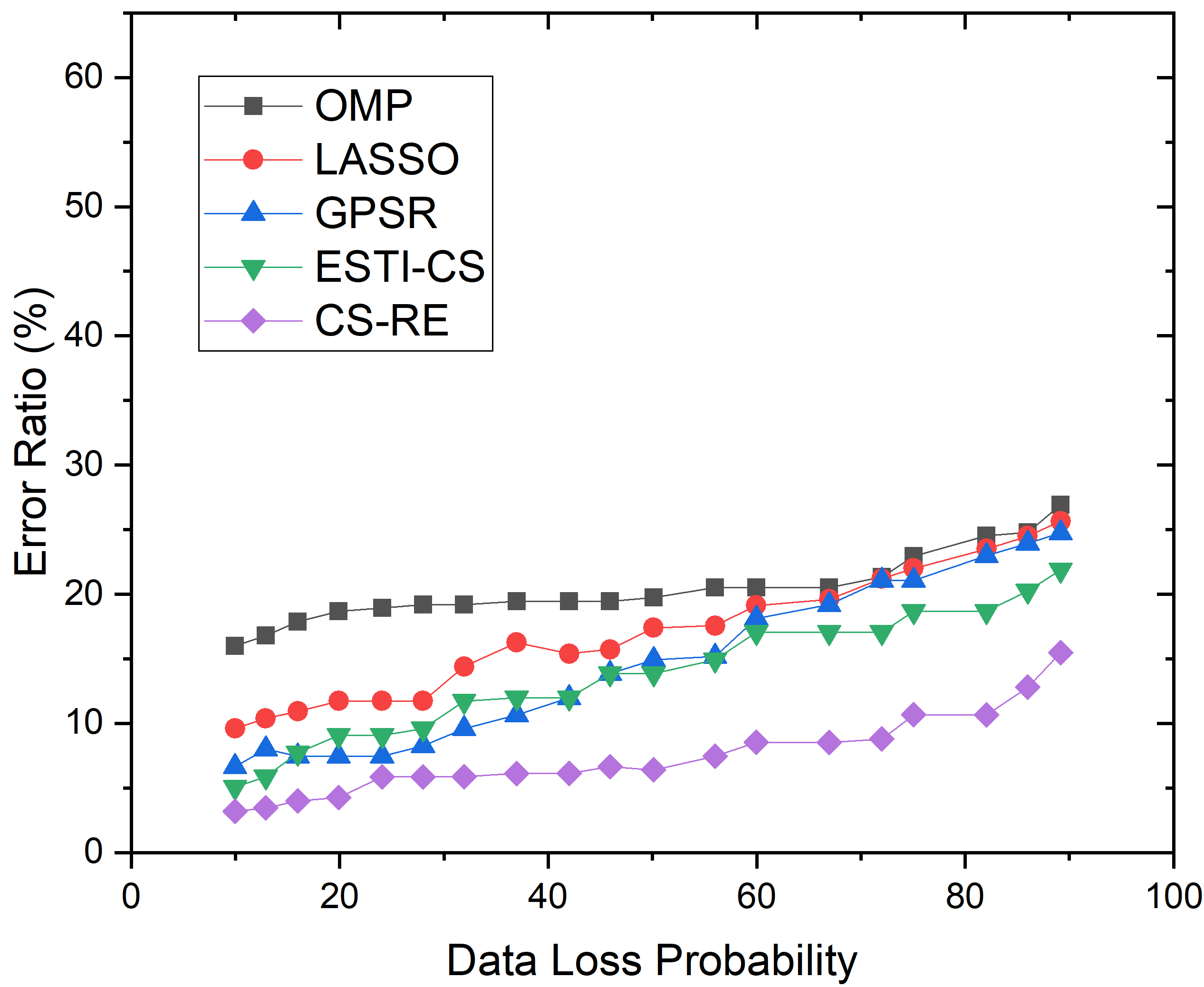

Supplement: S9 Fig — This figure shows the error ratio comparison of out CS-RE method with ESTI-CS, GPSR, LASSO, and OMP considering indoor light from Intel dataset. (PNG) [file pone.0245847.s009.png]

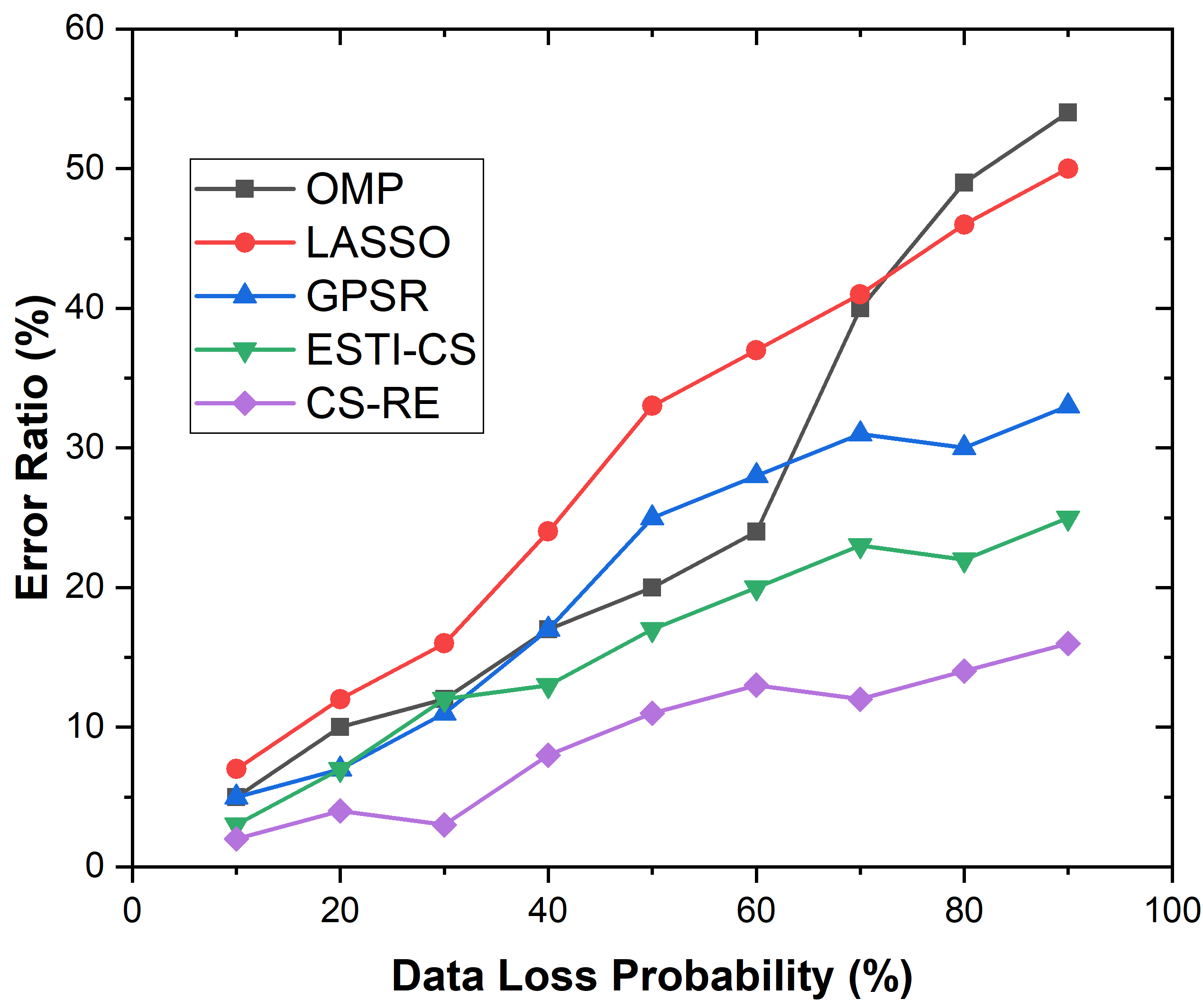

Supplement: S10 Fig — This figure shows the error ratio comparison of our CS-RE method with ESTI-CS, GPSR, LASSO, and OMP considering forest temperature from GreenOrbs project. (PNG) [file pone.0245847.s010.png]

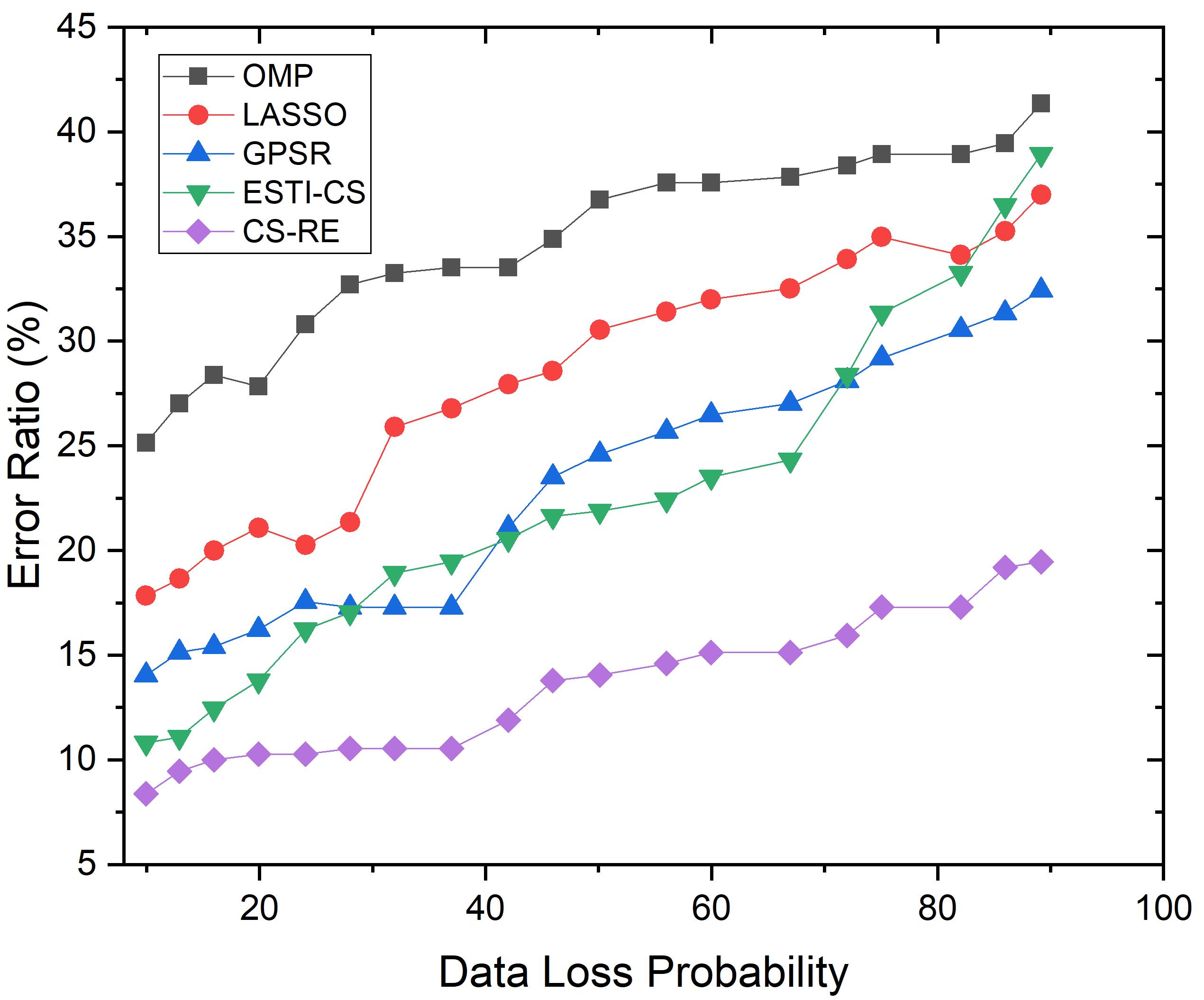

Supplement: S11 Fig — This figure shows the error ratio comparison of our CS-RE method with ESTI-CS, GPSR, LASSO, and OMP considering forest light from GreenOrbs project. (PNG) [file pone.0245847.s011.png]

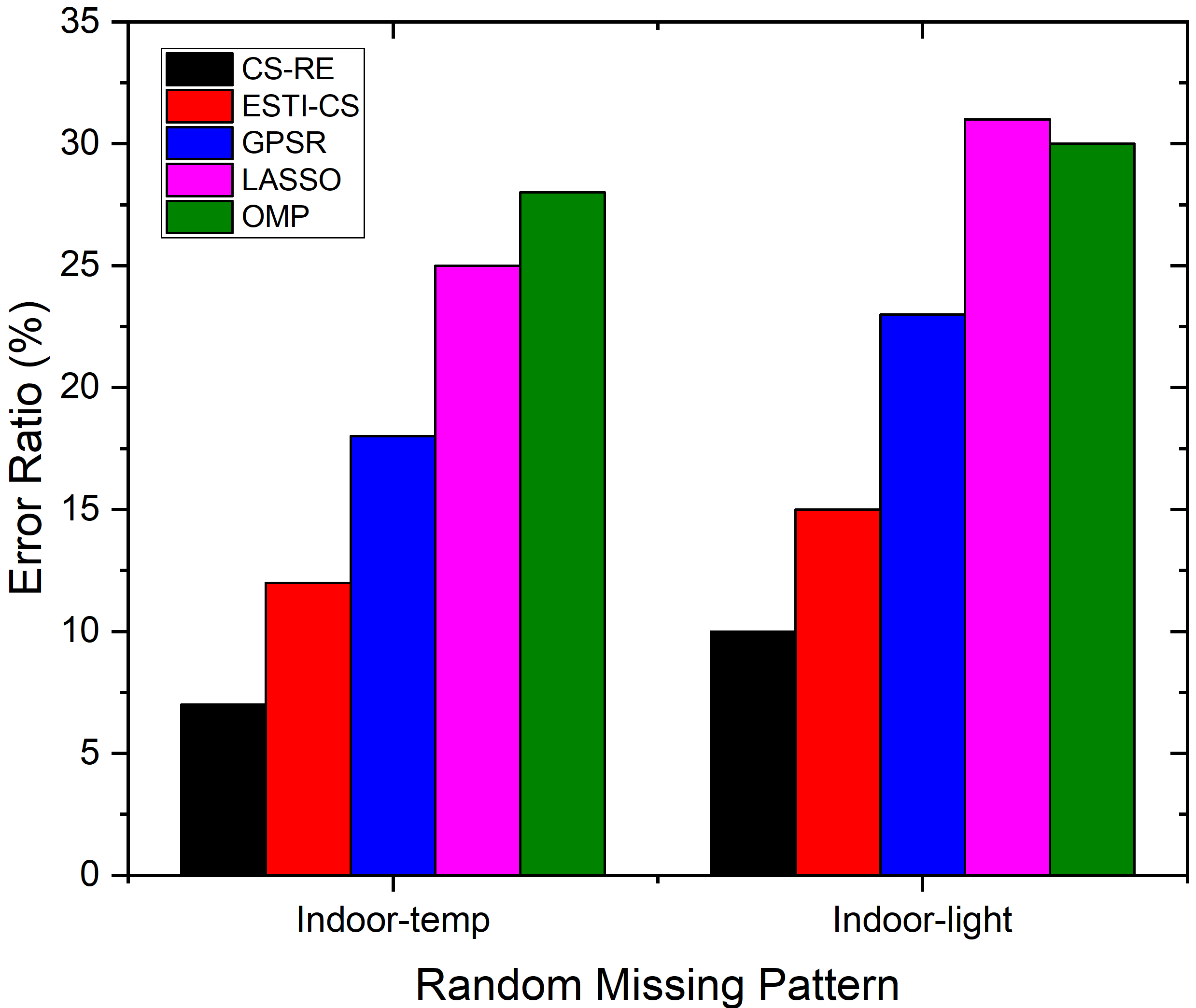

Supplement: S12 Fig — The figure shows the comparison histogram of random missing pattern using Intel dataset with 40% data loss. (PNG) [file pone.0245847.s012.png]

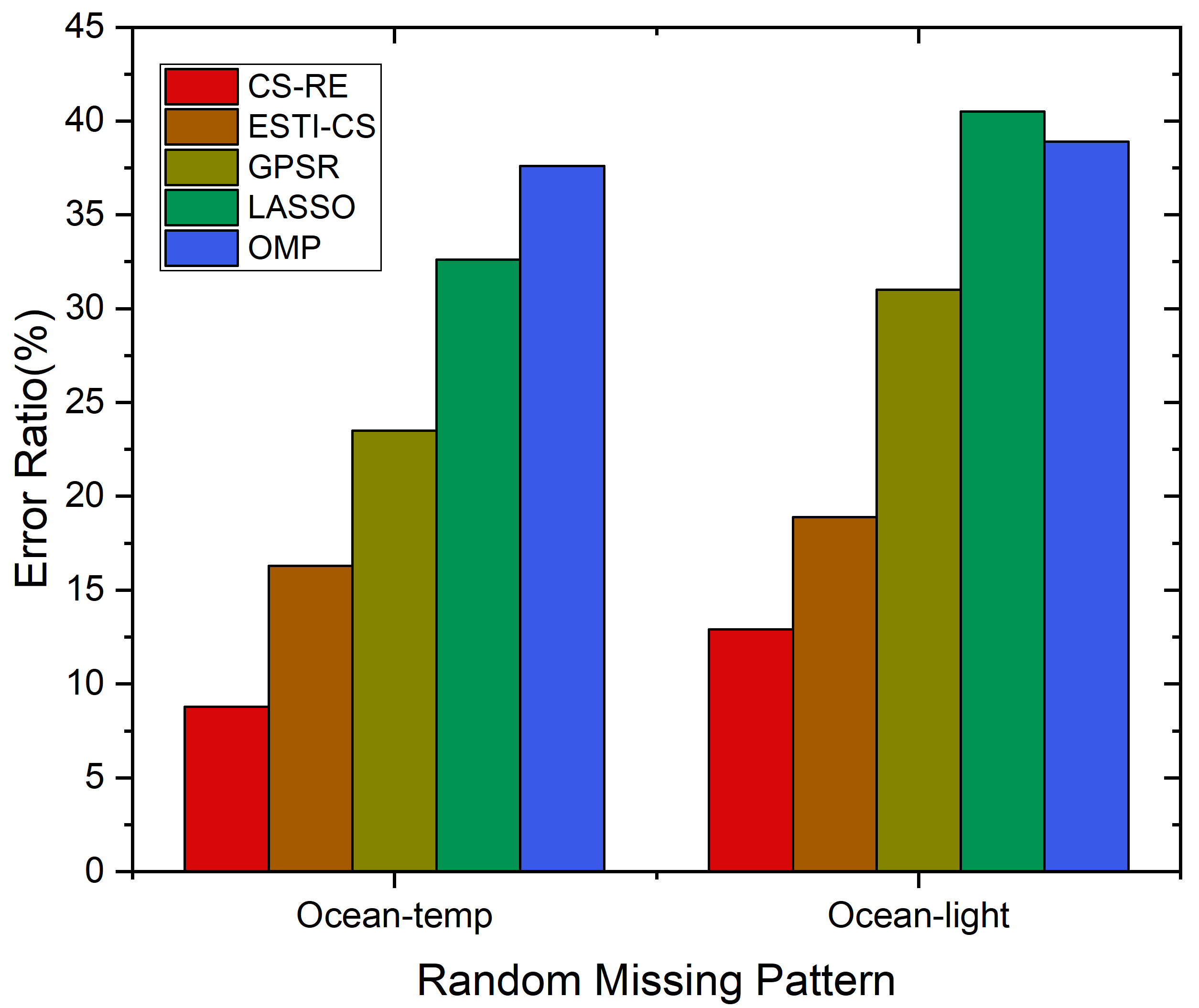

Supplement: S13 Fig — The figure shows the comparison histogram of random missing pattern using Ocean sense project dataset with 64% data loss. (PNG) [file pone.0245847.s013.png]

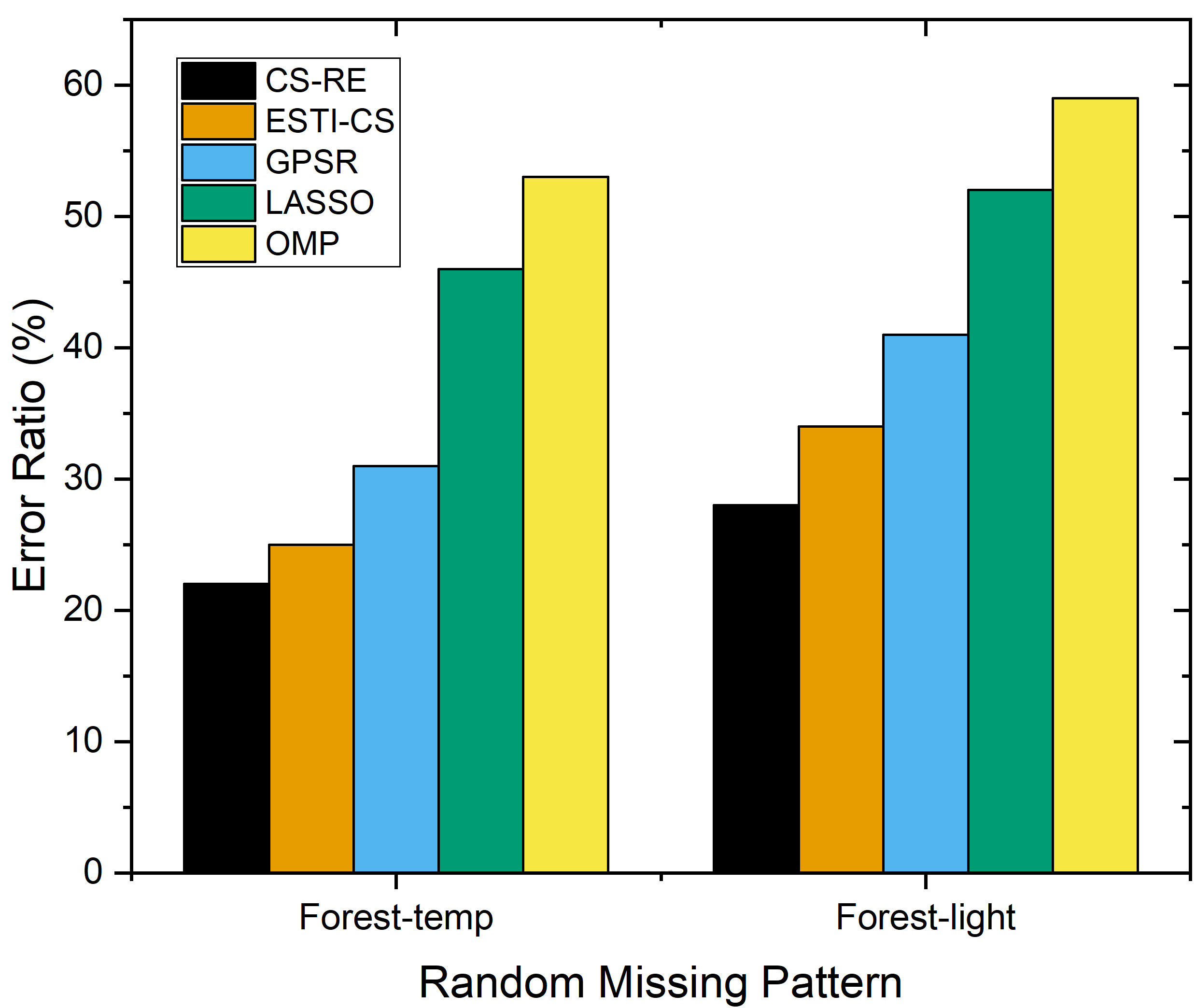

Supplement: S14 Fig — This figure shows the comparison histogram of random missing pattern using GreenOrbs project dataset with 30% data loss. (PNG) [file pone.0245847.s014.png]

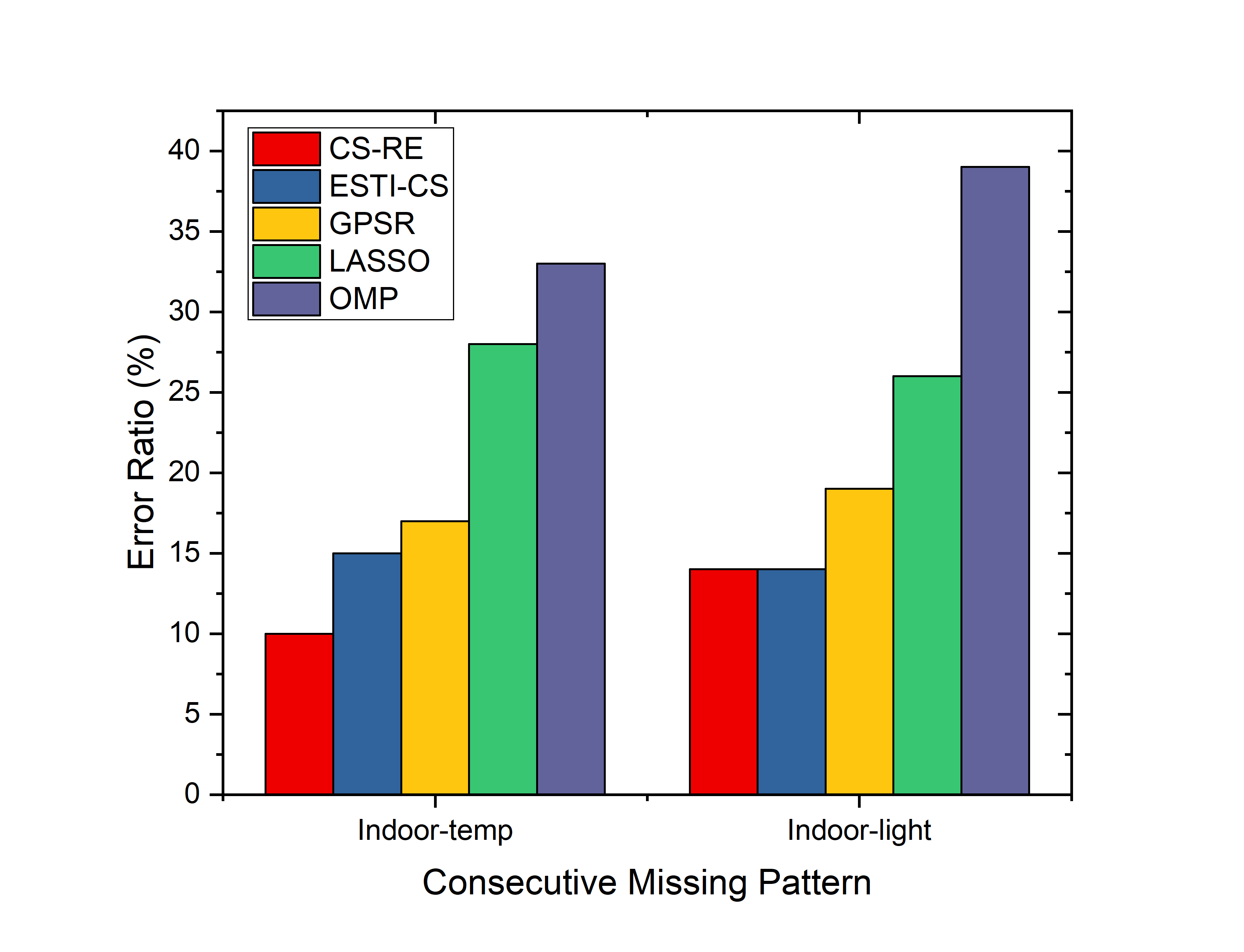

Supplement: S15 Fig — This figure shows the comparison histogram of our proposed methods with existing methods using consecutive missing pattern in Intel indoor dataset. Overall data loss is 40%. (PNG) [file pone.0245847.s015.png]

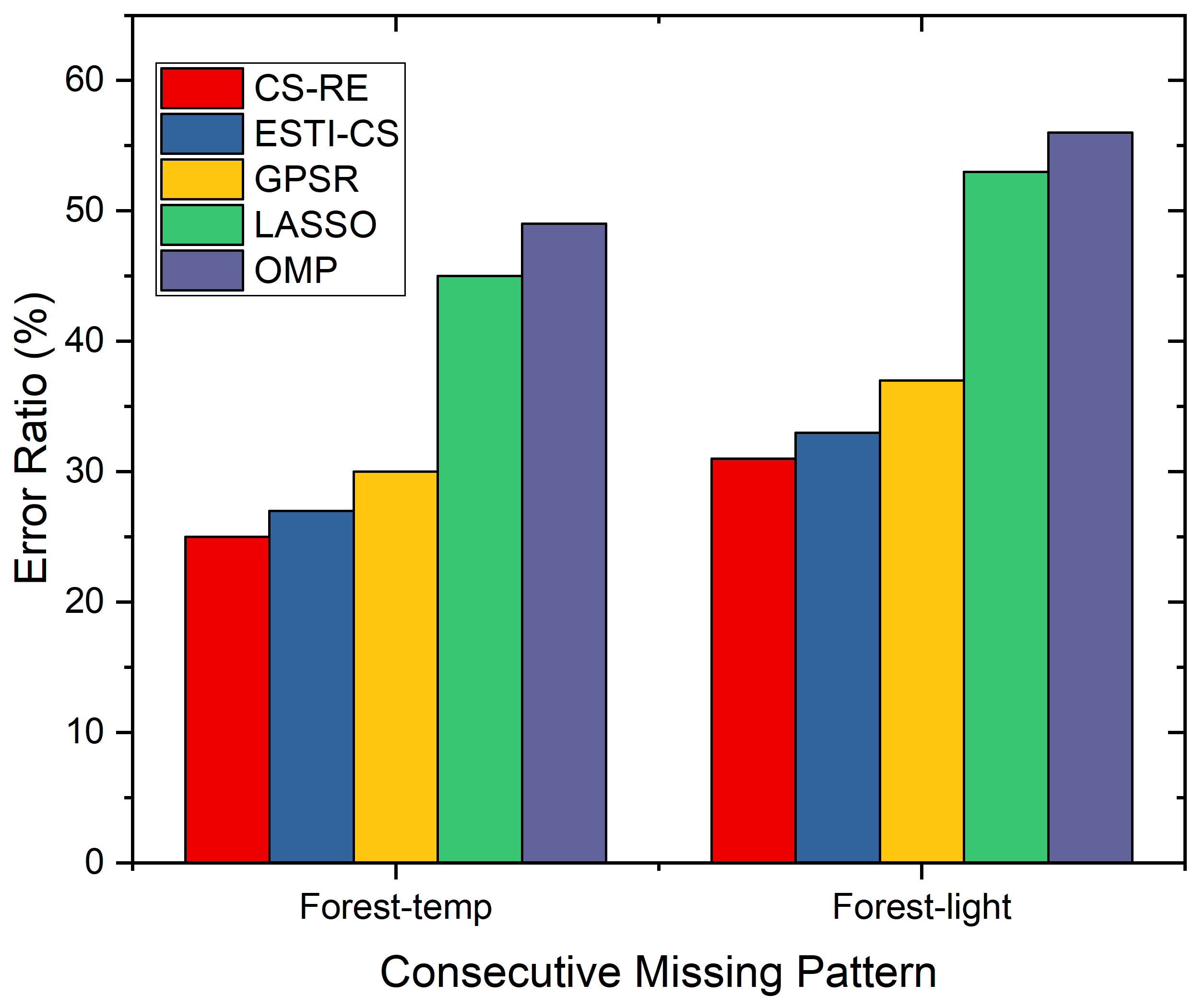

Supplement: S16 Fig — This figure shows the comparison histogram of our proposed methods with existing methods using consecutive missing pattern in GreenOrbs project dataset. Overall data loss is 30% here. (PNG) [file pone.0245847.s016.png]

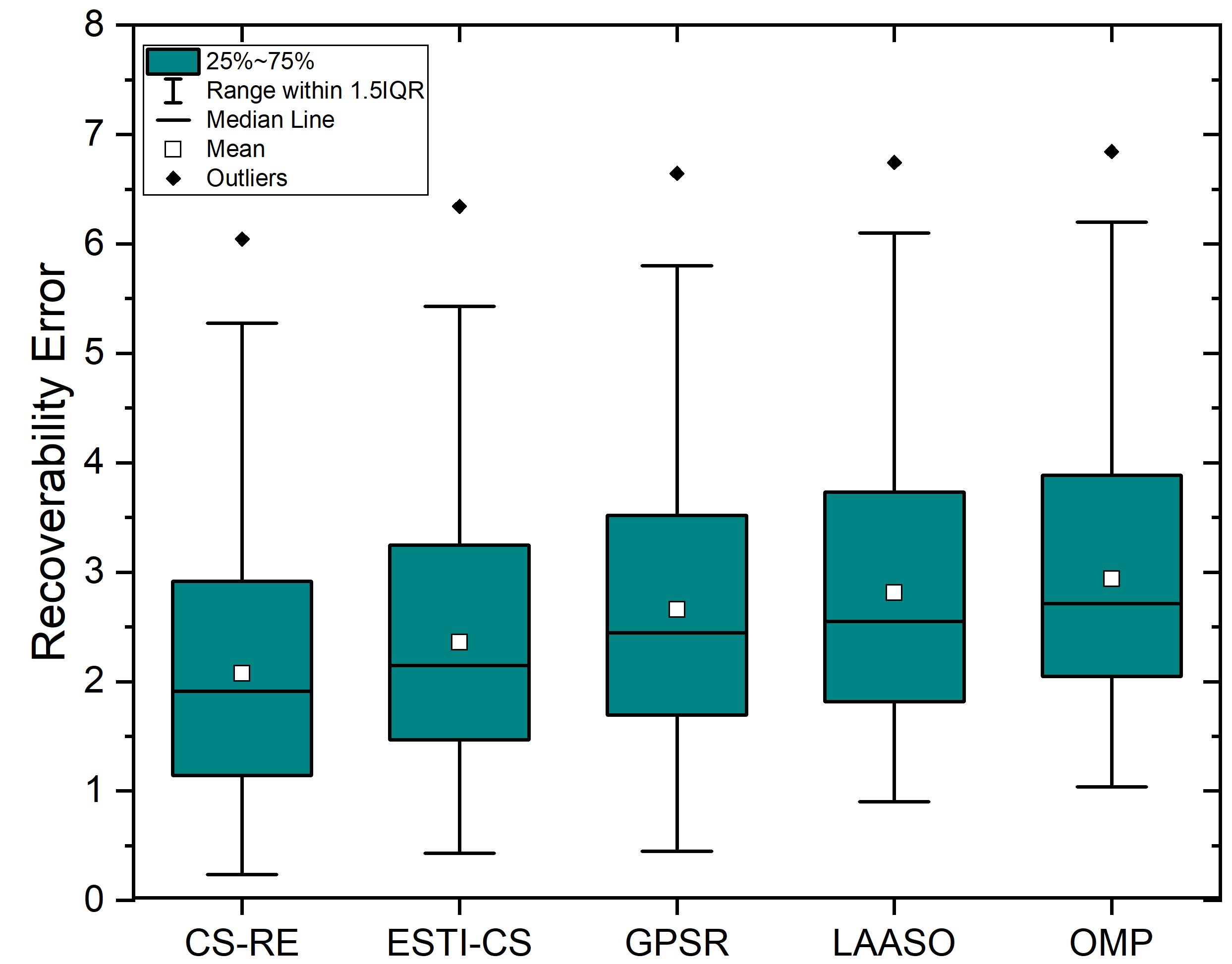

Supplement: S17 Fig — This figure shows the comparison of our proposed method with other methods in terms of reconstruction errors using Intel indoor dataset. (PNG) [file pone.0245847.s017.png]

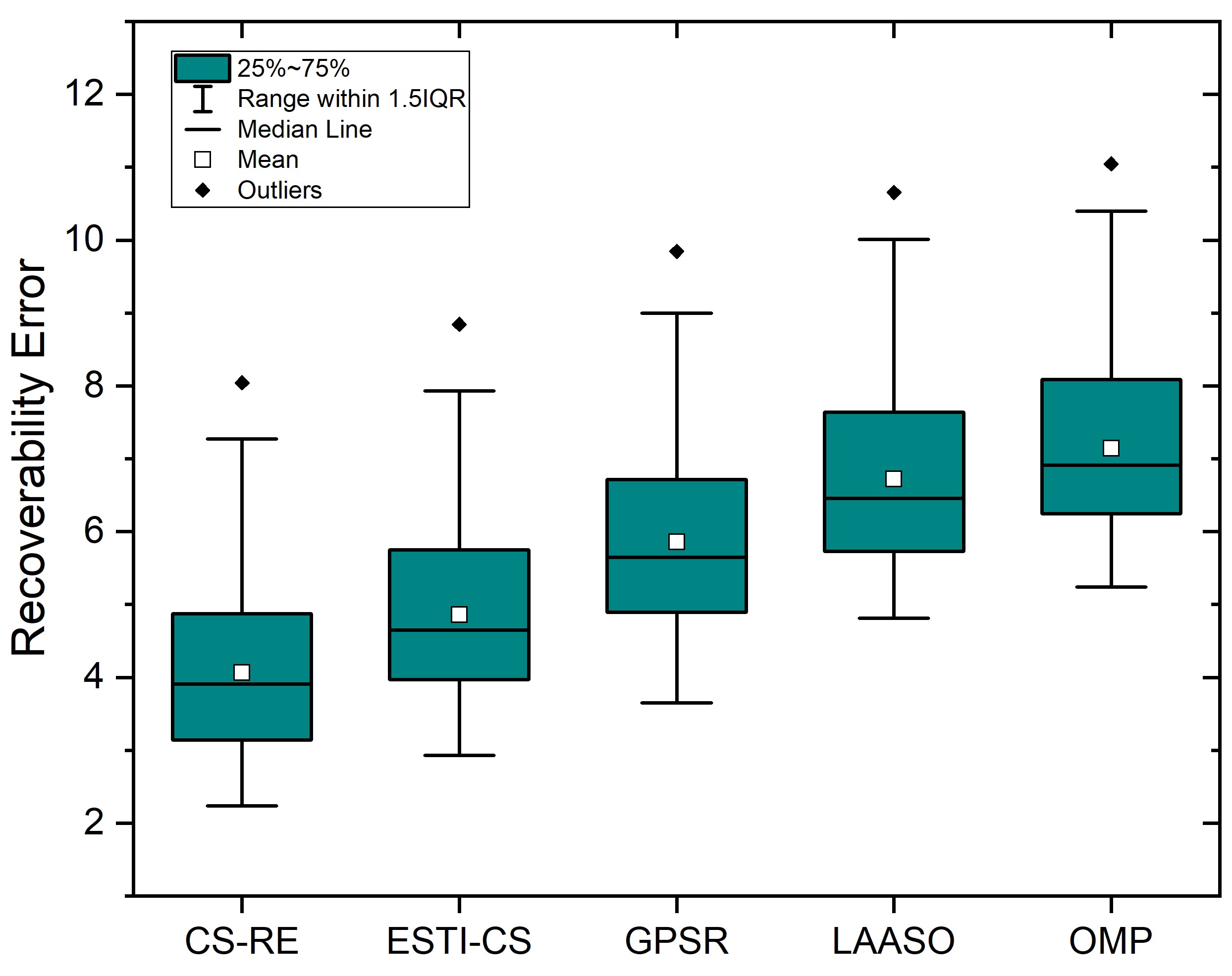

Supplement: S18 Fig — This figure shows the comparison of our proposed method with other methods in terms of reconstruction errors using GreenOrbs project dataset. (PNG) [file pone.0245847.s018.png]

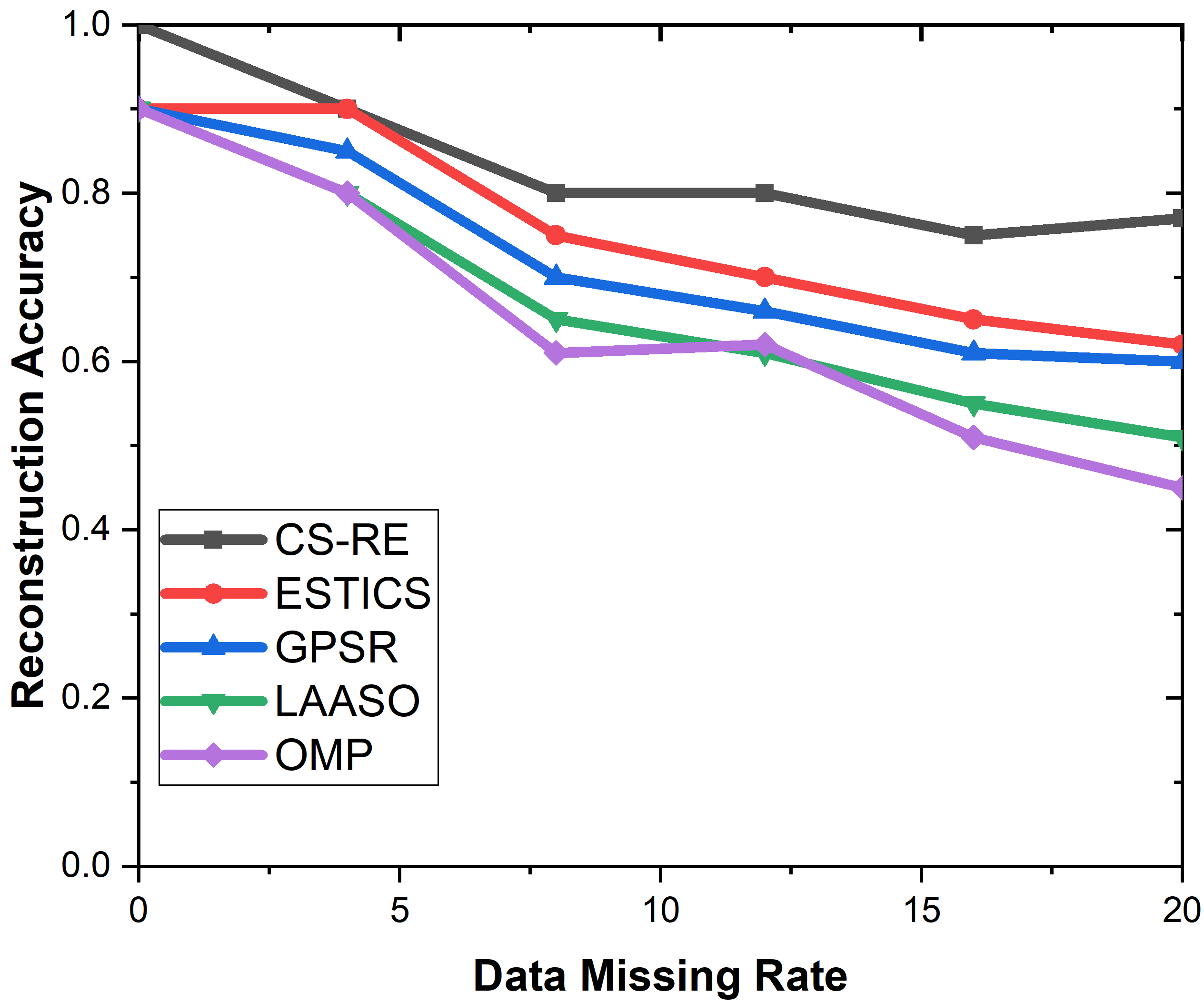

Supplement: S19 Fig — This figure shows the reconstruction accuracy of our proposed method as compared to other methods under consideration using Intel indoor dataset. (PNG) [file pone.0245847.s019.png]

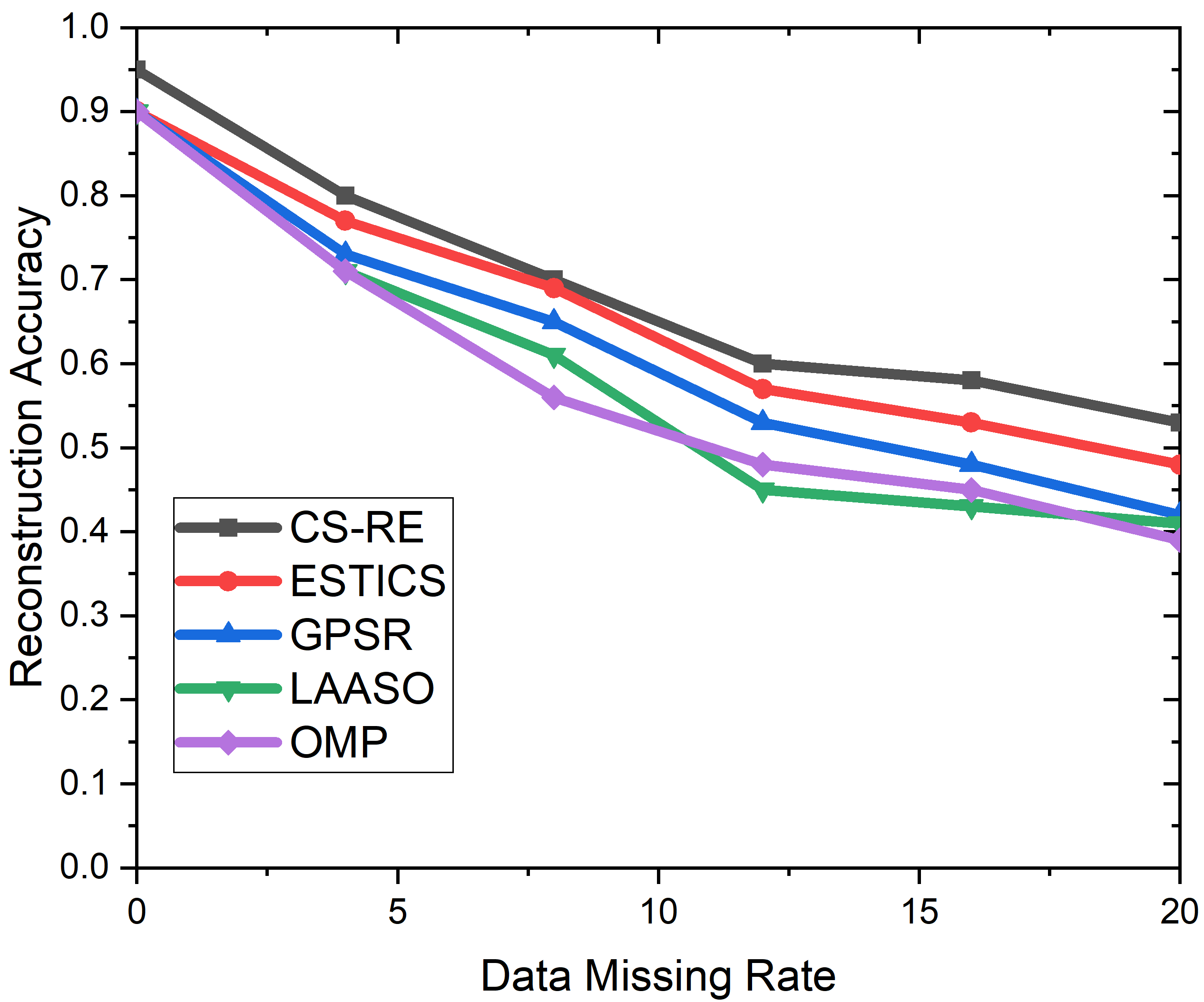

Supplement: S20 Fig — This figure shows the reconstruction accuracy of our proposed method as compared to other methods under consideration GreenOrbs project dataset. (PNG) [file pone.0245847.s020.png]
